# Flavoglycans: Evidence for Covalent Proanthocyanidin–Polysaccharide Linkages in Western Redcedar Bark

**DOI:** 10.1021/acs.jafc.6c03146

**Published:** 2026-09-10

**Authors:** Gio Ferson M. Bautista, Oliver Musl, Markus Bacher, Zhicheng Xia, Julia L. Azzi, Antje Potthast, Thomas Rosenau, Orlando J. Rojas

**Affiliations:** † Department of Chemistry, 8166 The University of British Columbia, 2036 Main Mall, Vancouver, BC V6T 1Z1, Canada; ‡ Bioproducts Institute, The University of British Columbia, 2385 East Mall, Vancouver, BC V6T 1Z4, Canada; § Department of Natural Sciences and Sustainable Resources, Institute of Chemistry of Renewable Resources, BOKU University, Konrad-Lorenz-Strasse 24, 3430 Tulln, Austria; ∥ Department of Chemical and Biological Engineering, The University of British Columbia, 2360 East Mall, Vancouver, BC V6T 1Z3, Canada; ⊥ Department of Wood Science, The University of British Columbia, 2424 Main Mall #2900, Vancouver, BC V6T 1Z1, Canada

**Keywords:** cell wall, tannins, crosslinking, polyphenols, *C*-glycosylation, flavonoids

## Abstract

We investigated covalent linkages between proanthocyanidins (PAs) and cell wall polysaccharides in western redcedar bark (*Thuja plicata* Donn), a tannin-rich forestry co-product that has potential as a functional food ingredient. An alcohol-insoluble residue (AIR; 1.0% w/w relative to oven-dried bark) recovered from aqueous extracts contained both PAs (≥1.46% w/w of AIR) and polysaccharides (∼30% w/w of AIR) and resisted fractionation by inert solvents, consistent with recalcitrant PA–carbohydrate association. Acidic butanol hydrolysis coupled with LC–MS revealed diagnostic *C*-glycosylated anthocyanidins, including 6-*C*-pentosylcyanidin, 8-*C*-(deoxyhexosyl)­cyanidin, and X-*C*-(deoxyhexosyl)-Y-*C*-pentosyldelphinidin. NMR analysis supported the presence of PA–polysaccharide conjugates, implicating hemicelluloses and pectins as the carbohydrate partners. These “flavoglycans” provide a chemical basis for incomplete tannin extractability from bark matrices and suggest that carbohydrate conjugation can modulate PA accessibility and interfacial interactions in aqueous systems relevant to bioactive recovery from bark matrices. Overall, this research provides converging lines of experimental evidence suggestive of *C*-glycosyl crosslinking between proanthocyanidins and carbohydrates in bark.

## Graphical abstract

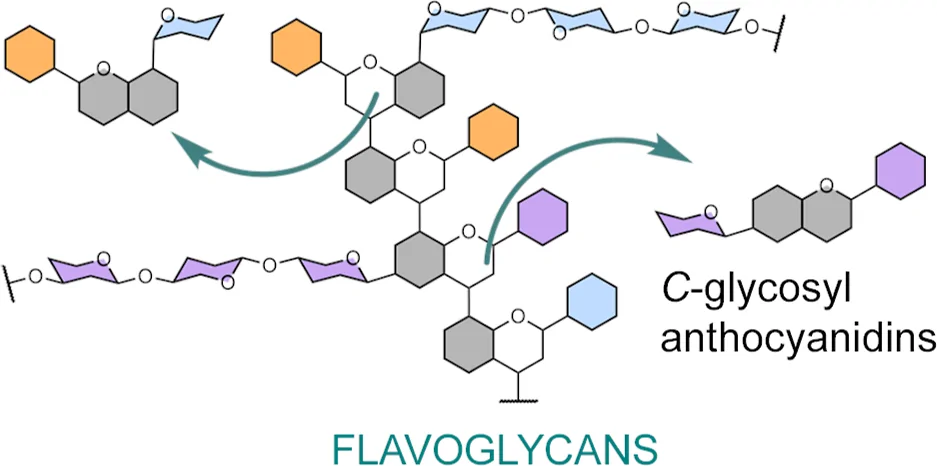

## 1 Introduction

The plant cell wall is a complex composite composed of cellulose, hemicelluloses, pectic polysaccharides, glycoproteins, and a variety of polyaromatic structures.^1^ The extent of occurrence and the mechanisms underlying polyphenol crosslinking within the cell wall matrix remain incompletely understood. Over the past decades, substantial progress in proanthocyanidin research has provided new perspectives on the interactions between proanthocyanidins and plant cell walls, including both non-covalent associations and covalent crosslinking.^2^ Moreover, non-covalent interactions have been revealed in apple cell wall material and purified polysaccharides studied in vitro.^3–5^ Significant information is also available on the interactions between grape proanthocyanidins and cell walls.^6,7^ In contrast, covalent crosslinking of proanthocyanidins to cell wall polysaccharides has been less frequently reported and remains poorly understood.

In general, covalent bonds involving proanthocyanidins may form through the oxidation of their catechol moieties (B-ring) to quinone or semiquinone. These subsequently react with nucleophilic thiol, amine, or alcohol groups, to form a covalent bond.^8^ In addition, nucleophilic attack of the phloroglucinol-type A-ring of proanthocyanidins on activated carbonyl compounds has been proposed as an alternative reaction pathway.^9^ Only a limited number of studies have reported covalently linked proanthocyanidin-polysaccharide structures,^10–13^ while several others provide indirect evidence for such linkages.^14–16^ Although the precise nature of covalent bonding between polysaccharides and proanthocyanidins remains an open question in plant polyphenol chemistry, extensive knowledge exists regarding *O-* and *C-*glycosylation patterns of monomeric flavonoids.^17–22^ These established glycosylation motifs offer important clues for understanding potential covalent glycosylation patterns in oligomeric and polymeric proanthocyanidins.

The glycosylation of proanthocyanidins as a mechanism for their association with the plant cell wall was first proposed by Porter et al. in 1985. This was based on the isolation of procyanidin *O-*β*-*glucopyranosides from quince fruits as well as spruce and pine barks.^23^ Neilson et al. isolated proanthocyanidin glycoconjugates (flavoglycans) from mangrove leaves and provided further evidence for covalent linkages between high-molecular-weight polysaccharides and flavan-3-ol polymers.^11^ Since then, several investigations have reported the widespread occurrence of mono-*O-*glycosides in proanthocyanidin extracts.^14,16,22,24,25^ In contrast, mono-*C-*glycosylated proanthocyanidins have been reported far less frequently, typically at trace (ppm level) abundances.^18,26^

In this regard, aqueous extracts of WRC bark have been reported to contain both proanthocyanidins (∼4–7%) and polysaccharides,^27^ and their co-occurrence have historically complicated extract utilization. As part of our ongoing efforts to understand the chemical composition of WRC bark extracts, we have undertaken a detailed structural characterization of the polysaccharides and proanthocyanidins present in the alcohol-insoluble residue (AIR).^28^ In our previous work, we encountered persistent difficulties in separating polysaccharide and proanthocyanidin components of the AIR. This suggests the involvement of multiple, cooperative non-covalent interactions as well as the possible presence of covalent linkages.

In our previous study, thiolysis of WRC bark AIR was used to determine proanthocyanidin monomer subunits.^28^ However, identification of *O*- or *C*-glycosylated units was not attainable by this methodology. The relatively mild conditions used in thiolysis also led to a low recovery as recalcitrant polysaccharides prevented effective digestion of the sample.

In contrast, acidic butanol hydrolysis is an oxidative depolymerization technique commonly used for proanthocyanidin analysis. This reaction yields anthocyanidins that absorb strongly at approximately 520 nm.^29^ In contrast to thiolysis, acidic butanol hydrolysis has been reported to be more effective for depolymerizing covalently modified proanthocyanidins, including glycosylated species.^30^ The elevated temperature and acid concentration employed in acidic butanol hydrolysis are thought to promote more extensive depolymerization of associated polysaccharides. These conditions are especially effective for the cleavage of less stable furanoses and deoxyhexoses,^31^ thereby increasing accessibility of proanthocyanidins to oxidative cleavage.

Under acidic butanol hydrolysis conditions, *O*-glycosidic bonds are readily cleaved,^32^ and proanthocyanidin *O*-glycosides therefore yield the corresponding anthocyanidins (aglycons or butyl glycosides). In contrast, proanthocyanidin *C*-glycosides resist acid-catalyzed cleavage and remain intact throughout the reaction.^33^ This selectivity greatly facilitates the identification of *C*-glycosylated anthocyanidins by LC–MS, providing structural information on the parent proanthocyanidins. To the best of our knowledge, this approach has not previously been applied to investigate *C-*glycosylation in proanthocyanidins.

This study investigates the covalent attachment of polysaccharide chains to proanthocyanidins through *C-*glycosylation. Previously unconfirmed interpolymer linkages were investigated using complementary nuclear magnetic resonance (NMR) spectroscopy and liquid chromatography–mass spectrometry (LC–MS) analysis of acidic butanol hydrolysis products. Finally, based on the experimental evidence obtained, we propose the first tentative structural model for flavoglycans in western redcedar bark.

## 2 Experimental Section

### 2.1 Chemicals and Materials

The stem bark of Western redcedar (*Thuja plicata* Donn) was provided by the Teal Jones Group sawmill (Surrey, BC V4N 4M8, Canada). The bark originated from trees felled in Terrace, BC and transported on the Fraser River before being debarked at the Surrey sawmill. The bark was air-dried (9.28 ± 0.18% moisture content) and ground to pass a 10-mesh screen prior to aqueous extraction.

Anthocyanidin standards, including cyanidin, delphinidin, peonidin, petunidin, malvidin, and pelargonidin, were purchased from Extrasynthese (Lyon, France). Flavan-3-ol standards included epicatechin (Sigma-Aldrich, St. Louis, MO, USA), procyanidin B2, and procyanidin B3 (Extrasynthese, Lyon, France). Monosaccharides, disaccharides, polysaccharides, and other carbohydrate standards, including l-arabinose, d-galactose, d-glucose, d-xylose, d-mannose, l-rhamnose, l-fucose, d-glucuronic acid, d-galacturonic acid, d-sorbitol, cellobiose, native wheat starch, Avicel PH-101, citrus peel pectin, and birchwood xylan, were obtained from Sigma-Aldrich (St. Louis, MO, USA). Additional carbohydrate standards included 4-*O*-methyl-d-glucuronic acid (Biosynth, Staad, Switzerland), isoprimeverose (AABlocks, San Diego, CA, USA), β-glucan (barley, high viscosity; Megazyme, Bray, Ireland), xylobiose, and tamarind gum (xyloglucan) (TCI, Tokyo, Japan). Stationary phases, including Sephadex LH-20, Dowex 50W-X8, Amberlite XAD7HP, insoluble polyvinylpolypyrrolidone (PVPP), and Silica Gel 60 G, were purchased from Sigma-Aldrich (St. Louis, MO, USA).

### 2.2 Preparation of Alcohol-Insoluble Residue from Bark

AIR was obtained according to our previous work.^28^ The bark was extracted with water at 20–25 °C for 16 h. To remove loosely bound components, the resulting aqueous extract was washed sequentially with 20 volumes of methanol followed by 75% (v/v) aqueous acetone. The resulting alcohol-insoluble residue (AIR) was stored at 4 °C, and aliquots were taken for subsequent analyses. A summary of this procedure is shown in Figure S1.

### 2.3 Depolymerization of AIR by Acidic Butanol Hydrolysis and Fractionation of its Products

Acidic butanol hydrolysis was performed following the procedure described by Porter et al.^29^ Approximately 500 mg of AIR was dispersed in 5 mL of 5% (v/v) concentrated HCl in *n*-butanol at 100 °C for 10 min and was rapidly cooled over ice. The mixture was centrifuged at 21,000 × *g* for 5 min and the supernatant was dried at room temperature using a vacuum concentrator (Savant Speed-Vac, ThermoScientific). The dried supernatant was redispersed in methanol/water/HCl (20/79.9/0.1 v/v) and filtered through a 0.45-μm nylon membrane filter. The filtrate was purified by solid phase extraction using an Amberlite XAD7HP column using the following eluents:^34^ (1) 0.1% (v/v) HCl-water to remove monomeric and oligomeric carbohydrates, (2) ethyl acetate to remove small neutral polyphenols, and (3) 0.1% (v/v) HCl–MeOH to elute the crude anthocyanin fraction.

For LC–UV/Vis-MS analysis, it was necessary to further separate anthocyanins from polymeric reaction products. For this, a mixture of 10% (w/w) insoluble PVPP-90% (w/w) and Silica Gel 60 G was prepared as stationary phase,^35^ and the concentrated crude anthocyanin fraction (3) was introduced onto the column. Anthocyanins (F1) were obtained by eluting the column with 0.1% (v/v) HCl–MeOH, and the polymeric reaction products (F2) were obtained by eluting with 50% (v/v) formic acid-MeOH. F1 was dried and redissolved in 1% v/v formic acid in methanol for further analysis by LC-MS (Supplementary Methods). Chromatograms and mass spectra were processed using MestReNova V15.0.1 software (A Coruña, Spain). For each peak of interest, the average mass spectrum was background-subtracted prior to analysis. Peaks detected at 520 nm were integrated and quantified by single-point calibration using authentic anthocyanidin standards (delphinidin, cyanidin, pelargonidin, and peonidin). *C*-glycosylated anthocyanidins were quantified at 520 nm and are reported as cyanidin equivalents. For the presumed chalcone form, absorbance at 280 nm was used to estimate the corresponding cyanidin equivalent. All quantitative data are based on single chromatographic analyses of both the standards and the samples.

Various mixtures of flavan-3-ols (monomers and dimers) and carbohydrate standards (monosaccharides, disaccharides, and polysaccharides) were prepared and subjected to acidic butanol hydrolysis (Table S1) to evaluate the potential in situ formation of *C*-glycosides during hydrolysis. Briefly, 5 μL of a 10 mmol/L flavan-3-ol solution in methanol was combined with 10 mg of carbohydrate standard (corresponding to an approximate 1:1 molar ratio of monosaccharide to flavan-3-ol monomer). Subsequently, 200 μL of the acidic butanol reagent was added, and the reaction mixture was heated at 100 °C for 10 min. After centrifugation of the cooled mixture, the supernatant was dried using a centrifugal evaporator, reconstituted in 50 μL of 1% (v/v) formic acid in methanol, and the reaction products were analyzed by LC–MS.

### 2.4 Determination of *C*-Glycosylation by NMR Spectroscopy

For NMR analysis, a larger amount of depolymerized WRC bark AIR was required. Approximately 4.5 g sample was depolymerized in 50 mL of 5% (v/v) concentrated HCl in *n*-butanol under reflux at 100 °C in batches of timed intervals (10 min, 30 min, 1, 2, and 4 h), with the depolymerizing reagent replaced with fresh solution at the indicated times. The supernatant from each batch was pooled and processed as above. F1 was additionally purified on a Sephadex LH-20 column using a gradient of methanol/water/HCl from 20/79.9/0.1 v/v to 40/59.9/0.1 v/v. A broad band of pink compounds was determined visually and was collected and dried in a rotary evaporator (Buchi Rotavapor R220 Pro).

For sample preparation, a total of 8.4 mg of the purified F1 was dissolved in 65 μL of trifluoroacetic acid-*d* in dimethyl sulfoxide-*d*_*6*_ (1:9 v/v) and transferred into a 1.7 mm NMR tube for NMR analysis.

NMR spectra were recorded on a Bruker Avance 600 (resonance frequencies 600.15 MHz for ^1^H, 150.92 MHz for ^13^C) equipped with a 1.7 mm CPTCI ^1^H–^13^C/^15^N cryoprobe. Proton, ^1^H–^13^C heteronuclear single quantum coherence (HSQC), HSQC-total correlation spectroscopy (HSQC-TOCSY) with spin-lock time of 60 ms, ^1^H–^1^H correlation spectroscopy (COSY) and ^1^H–^13^C heteronuclear multiple bond correlation (HMBC) spectra of the sample were acquired at 303.5 K using the pulse sequences zg30, hsqcedetgp, hsqcdietgpsisp, cosygpqf and hmbcgplpndqf, respectively. The NMR spectra were processed and analyzed with MestReNova V15.0.1 software (La Coruña, Spain). The chemical shifts were referenced to the residual solvent signals for DMSO-*d*_6_ at (3.31 ppm for ^1^H, 49.15 ppm for ^13^C).

## 3 Results and Discussion

### 3.1 Isolation and Detection of *C*-Glycosylanthocyanidins from AIR

In our previous study,^28^ an alcohol-insoluble residue (AIR) was obtained from an aqueous extract of western redcedar bark. The AIR contained a substantial fraction of polysaccharides, as determined by acidic depolymerization (30 ± 1% w/w), including pectins, xyloglucans, and xylans. The proanthocyanidin content of AIR, determined by thiolysis, was 1.46 ± 0.04% (w/w) and consisted of oligomeric species with a mean degree of polymerization (mDP) of 5.3 ± 0.2 and an overall cis/trans ratio of 0.40 ± 0.04. The proanthocyanidins were composed primarily of catechin units (65 ± 3 mol %), with a procyanidin-to-prodelphinidin (PC/PD) ratio of 3.90 ± 0.83. The quantitative composition of AIR, as reported in our previous study,^28^ is reproduced in Tables S1 and S2 for reference.

Acidic butanol hydrolysis of analytical standards of model procyanidin dimers in the presence of model polysaccharides generally yielded the expected cyanidin product (Table S3). Importantly, no condensation products between procyanidin- or cyanidin-derived species and monosaccharides were detected. This indicates that glycosylated species observed after hydrolysis indeed originate from native structures present in AIR rather than being artifacts generated during the reaction. In addition to the expected cyanidin product, a later-eluting peak detected at 520 nm (Figure S2) was observed. The detected mass corresponds to the incorporation of a butyl group to the cyanidin skeleton (+56 Da; hypothetical structure shown in Figure S3). This suggests that butanol reacts with procyanidin intermediates or with the resulting anthocyanidin products during hydrolysis.

Butoxylated cyanidin species were also detected in the depolymerized WRC bark AIR. Most butoxylated products eluted at relatively late retention times in LC–MS (from ∼23 min onward). They overlap primarily with oligomeric species, particularly in the crude hydrolysate and fraction F2. Importantly, these anthocyanidin–butanol adducts did not co-elute with the anthocyanins of interest and therefore did not significantly interfere with chromatographic detection or identification of target anthocyanins and their aglycons. Additional support for butoxylation was obtained from NMR analysis of depolymerized WRC bark AIR. Heteronuclear single quantum coherence (HSQC) and heteronuclear multiple–bond correlation (HMBC) spectra revealed butoxyl moieties associated with anthocyanidins, anomeric carbons of carbohydrates (butyl glycosides, cf. formation of methyl glycosides upon acidic methanolysis of polysaccharides), and carboxyl groups in uronic acids (*i.e.,* butyl esters)^36^ as well as fatty acids (Figures 4 and 5).

**Figure 1:**
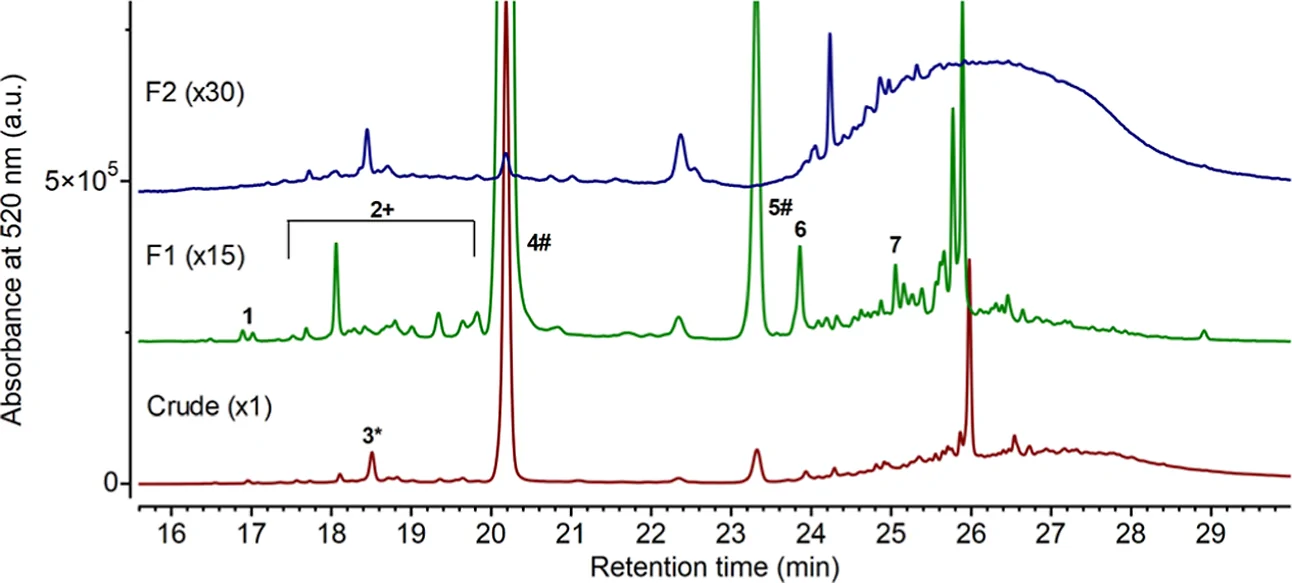
Acidic butanol hydrolysis of AIR. Scaling factors (in parentheses) have been used for comparison. Peaks 1,2, and 7 are the *C*-glycosylated anthocyanins. ^+^Group of peaks; *Delphinidin was only detected in the crude extract; ^#^Peak is to the left of the label.

**Figure 2:**
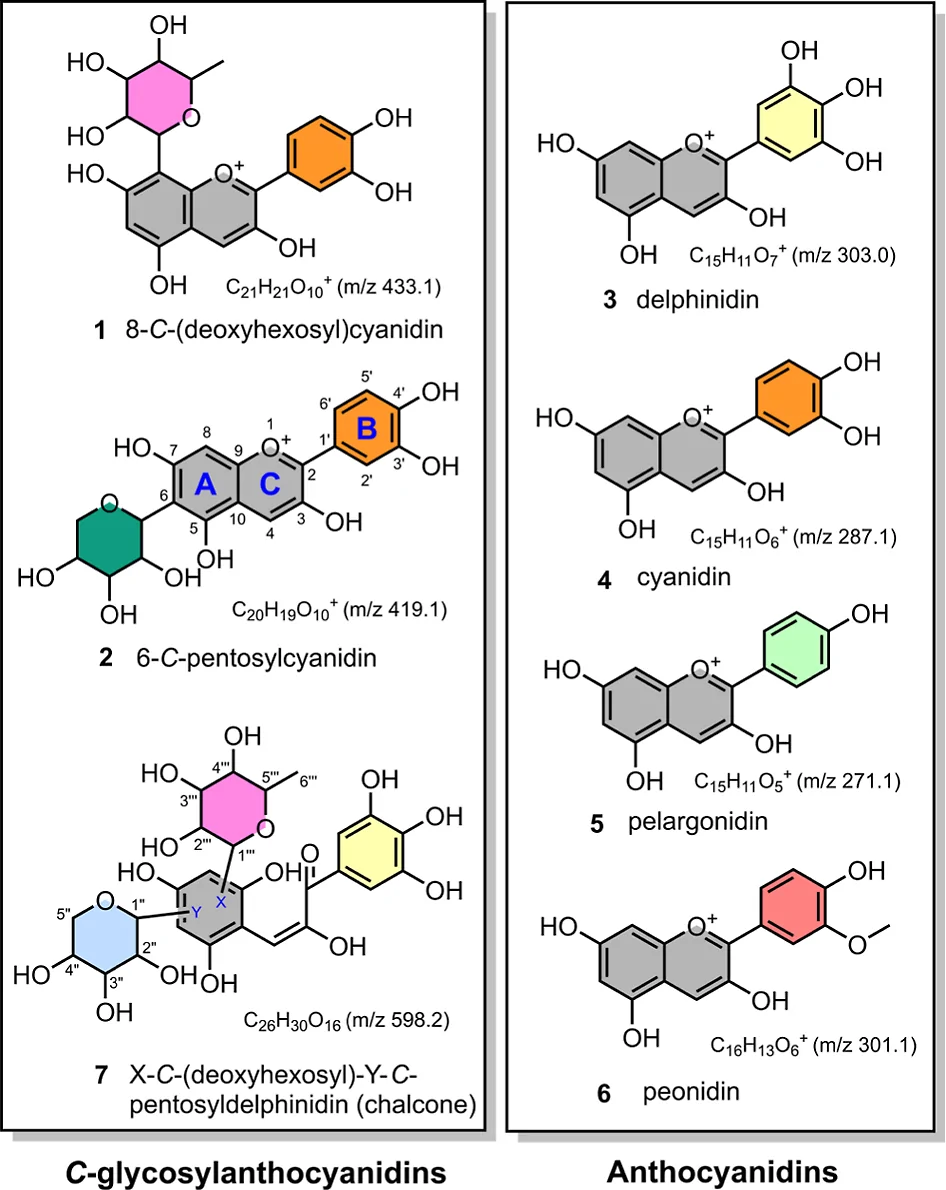
Anthocyanins and aglycons detected in AIR after acidic butanol hydrolysis.

**Figure 3:**
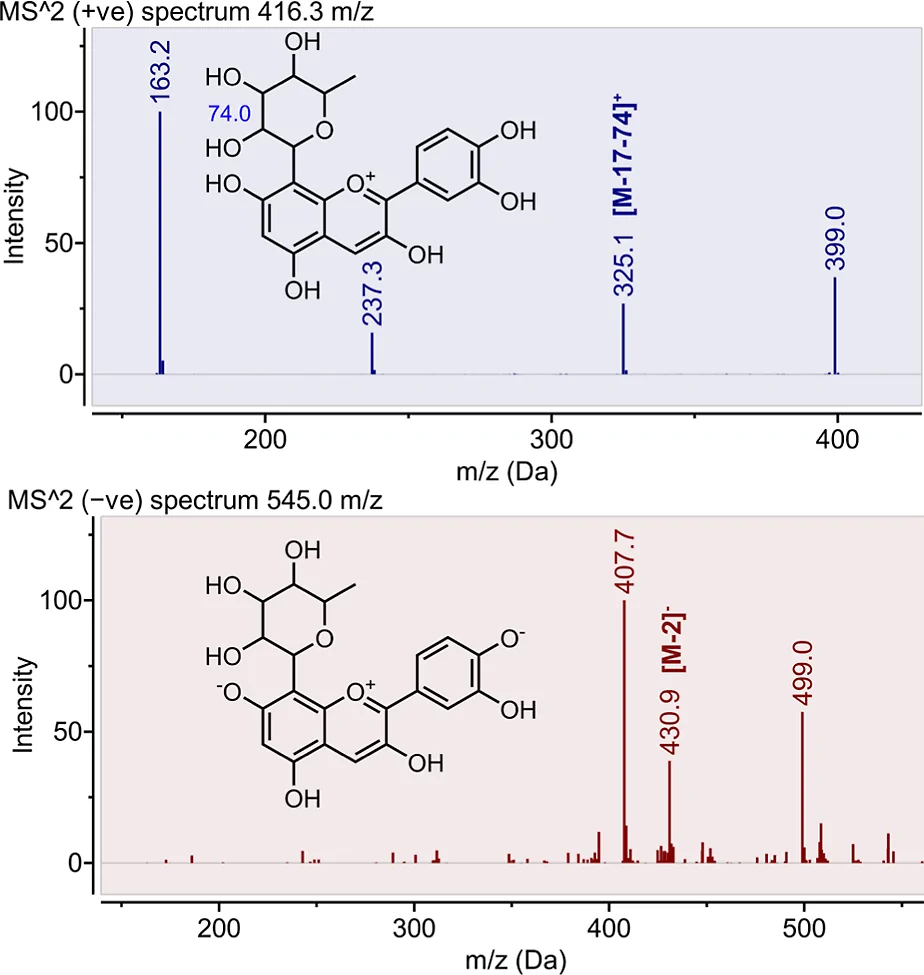
Tandem mass spectra of different precursor anthocyanins after acidic butanol hydrolysis of AIR. Positive (blue) and negative (red) ion mass spectra of 8-*C*-(deoxyhexosyl)­cyanidin (compound 1). Peak labels indicate the *m*/*z* observed, with the proposed mass losses annotated in bold.

**Figure 4:**
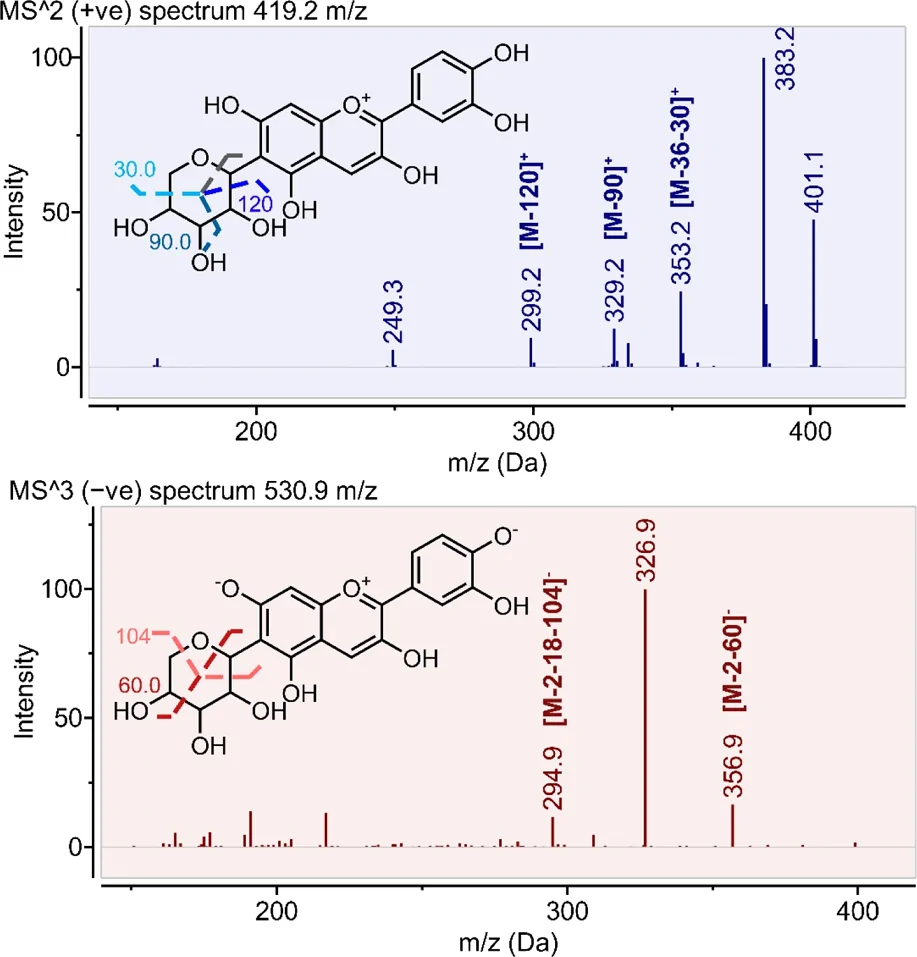
Tandem mass spectra of different precursor anthocyanins after acidic butanol hydrolysis of AIR. Positive (blue) and negative (red) ion mass spectra of 6-*C*-pentosylcyanidin (compound 2). Peak labels indicate the *m*/*z* observed, with the proposed mass losses annotated in bold.

**Figure 5:**
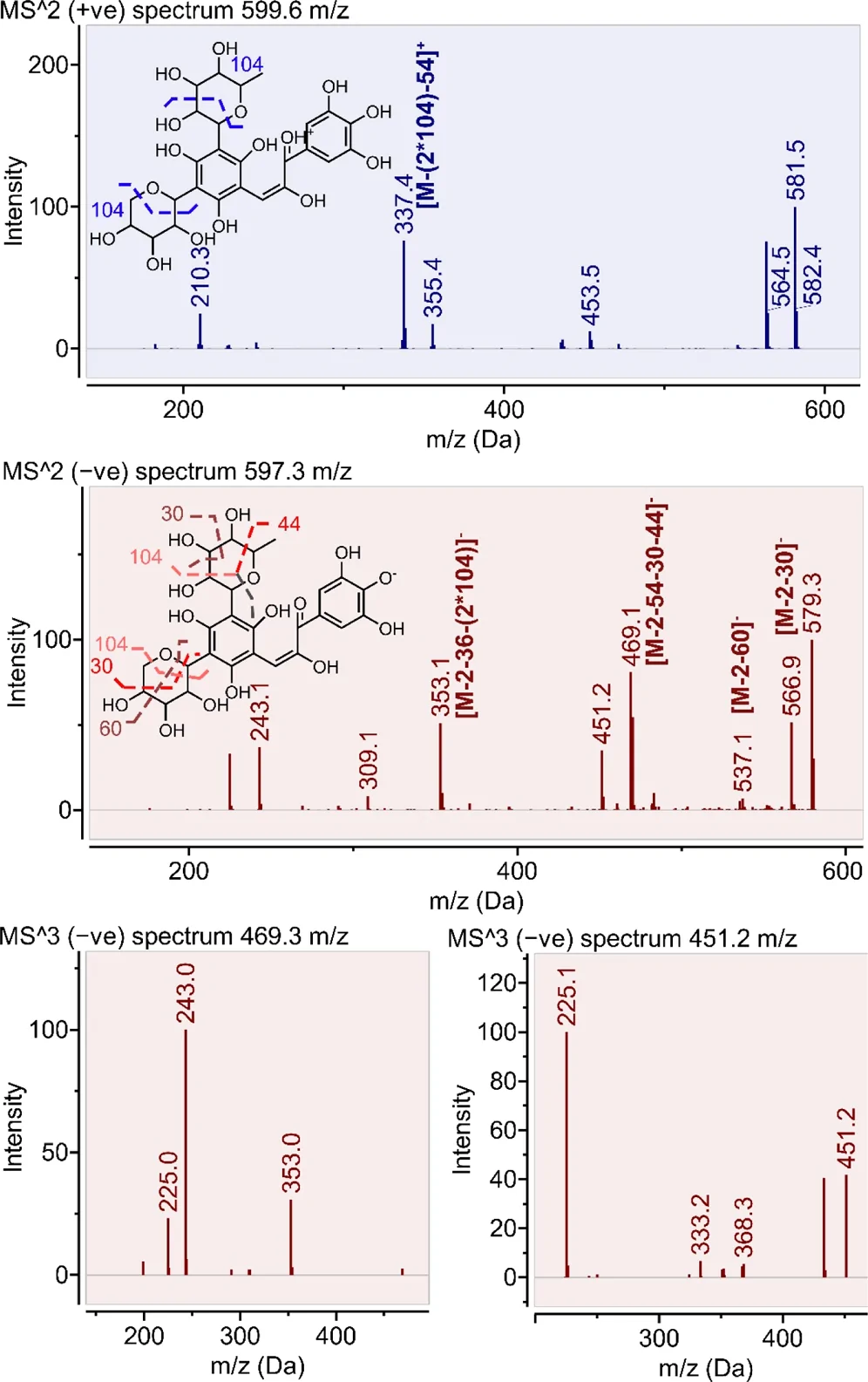
Tandem mass spectra of different precursor anthocyanins after hydrolysis of AIR. Positive (blue) and negative (red) ion mass spectra of X-*C*-(deoxyhexosyl)-Y-*C*-pentosyldelphinidin (compound 7). Peak labels indicate the *m*/*z* observed, with the proposed mass losses annotated in bold. For simplicity, the structures in the inset assume X = 8 and Y = 6.

Increasing either the HCl concentration or the hydrolysis time did not lead to improved resolution or higher intensity of discrete anthocyanidin peaks. Instead, broad signals indicative of polymeric reaction products were observed, likely arising from condensation reactions among anthocyanidins, proanthocyanidins, and flavan-3-ols.^37,38^ Such reactions consume free anthocyanidins and, under overly severe conditions, can suppress their detection entirely.^39^ Consequently, hydrolysis conditions were deliberately kept mild and short, followed by purification to remove polymeric reaction products that interfered with LC–MS analysis. Solid-phase extraction of the crude hydrolysate yielded a pinkish-red fraction enriched in anthocyanins and aglycons (F1) and a deep red fraction containing polymeric reaction products (F2). Owing to its polymeric nature and thus altered structure, fraction F2 was not subjected to further MS-based structural identification.

#### 3.1.1 Identification of Anthocyanins and Aglycons by Mass Spectrometry

A range of anthocyanidins were identified in fraction F1 by comparison with authentic reference standards (Figure S4), including cyanidin (compound 4), pelargonidin (compound 5), and peonidin (compound 6) (Figures 1 and 2 and Table 1). Delphinidin (compound 3) was detected only in the unpurified hydrolysate. The quantities of anthocyanidins were estimated using single-point calibration of the corresponding species at 520 nm as single measurements. The presence of cyanidin and delphinidin is consistent with our previous thiolysis results.^28^ In addition, the detection of peonidin at low abundance (∼2 × 10^–6^ mmol/g AIR, Table S4) indicates methylation at ring B of a subset of procyanidin units. This interpretation is supported by heteronuclear single quantum coherence (HSQC) (Figure 6B) and heteronuclear multiple–bond correlation (HMBC) (Figure 7B). The occurrence of methylated proanthocyanidins suggests that at least a fraction of these compounds may be deposited within the cell wall, as flavonoid methylation is commonly associated with cell wall-bound or immobilized flavonoid pools.^40^

**Table 1:** Retention Time, Absorbance, and MS Information for the Anthocyanins and Aglycons Detected after Fractionation of Acidic Butanol Hydrolysate

peak no.	compound	retention time (min)	λ_max_ (vis) (nm)	MS^*n*^ (positive) precursor and fragment ions (*m*/*z*)^a^	MS^*n*^ (negative) precursor and fragment ions (*m*/*z*)^a^
1	8-*C*-deoxyhexosylcyanidin	17.0	530	416.3 → 399.0, **325.1**, 237.1, 163.2	545.0 → 499.0, 430.9, 407.7
2	6-*C*-pentosylcyanidins	17.8–19.8	529	419.2 → 401.1, 383.2, **353.1**, 334.3, **329.2**, **299.2**, 249.3	530.9 → 416.9 → **356.9**, 326.9, **294.9**
3	delphinidin	18.5	510	303.2	415.0, 301.3
4	cyanidin	20.2	526	287.1	399.1, 285.4
5	pelargonidin	23.3	513	271.1	382.9 → 268.9
6	peonidin	23.9	530	301.3 → 286.3	412.9 → 298.9 → 283.9
7	X-*C*-(deoxyhexosyl)-Y-*C*-pentosyl-delphinidin (chalcone form)	25.1	330 (UV)	599.6 → 581.5, 563.5, 453.5, 355.4, **337.4**, 336.5, 210.3	597.3 → 579.3, **566.9**, **537.1**, **469.1**, 451.2, **353.1**, 309.1, 243.1, 225.1
					597.3 → 469.3 → 353.0, 243.0, 225.0
					597.3 → 451.2 → 433.2, 368.3, 333.2, 225.1

a Fragment ions highlighted in bold are characteristic of *C*-glycosylated anthocyanidins.

Identification of glycosylated anthocyanidins was based on tandem mass spectrometry experiments acquired in both negative and positive ion modes (Figures 3, 4, and 5). The proposed fragmentation pathways used for structural assignments are summarized in the Supporting Information. The compound eluting at 17.0 min was assigned as 8-*C*-(deoxyhexosyl)­cyanidin (compound 1). In negative ion mode, this species formed a trifluoroacetic acid (TFA) adduct at *m*/*z* 545.0, while in positive ion mode it exhibited a characteristic loss of 91 Da. This loss was attributed to ring cleavage of a *C*-bound deoxyhexosyl moiety (−74 Da) combined with the loss of a hydroxyl group (−17 Da), consistent with previously reported fragmentation behavior of *C*-glycosylated flavonoids^41^ and the common radical losses exhibited by anthocyanidins.^42^ While the expected [M]^+^ = 433 *m*/*z* for compound 1 was not observed due to possible in-source fragmentation, the TFA adduct observed in negative ion mode [M + TFA–2H]^−^ satisfies the expected *m*/*z* for this compound. The glycosylation position was assigned to C8 based on the absence of extensive ring fragmentation that are typical for 6-*C*-glycosyl moieties.

A cluster of peaks from compounds eluting between 17.8 and 19.8 min was assigned to epimeric sugar forms (xylose or arabinose) as well as different anomeric configuration (α or β) of sugar moieties in 6-*C*-pentosylcyanidin (compound 2). This assignment was supported by the observation of characteristic neutral losses of 60, 90, and 122 Da (corresponding to 104 + 18 Da) in negative ion mode, and losses of 66, 90, and 120 Da in positive ion mode. These fragmentation patterns are indicative of extensive ring cleavages associated with *C*-pentosyl substituents and are consistent with reported behavior of *C*-glycosylated flavonoids (Figure 4).^41^

The compound eluting at 25.1 min was assigned to X-*C*-(deoxyhexosyl)-Y-*C*-pentosyldelphinidin (compound 7) based on its complex fragmentation behavior. In negative ion mode, neutral losses of 30, 60, 128, and 244 Da were observed and attributed, respectively, to fragments associated with *C*-pentosyl or *C*-deoxyhexosyl moieties, a weak loss from a *C*-pentosyl moiety, a combined loss corresponding to *C*-pentosyl (30 Da), *C*-deoxyhexosyl (44 Da), and water (54 Da = 3 × 18), and a combined loss of *C*-deoxyhexosyl (140 Da) and *C*-pentosyl (104 Da) fragments. Larger product ions at *m*/*z* 451 and 469 underwent further fragmentation, yielding ions inconsistent with those expected for *O-*glycosylated species. On this basis, the presence of an *O*-deoxyhexosyl moiety was excluded for this compound.

In positive ion mode, a prominent neutral loss of 262 Da was observed, which can be rationalized either as the combined loss of a *C*-deoxyhexosyl fragment and water (158 Da = 104 + 3 × 18) together with a *C*-pentosyl fragment (104 Da), or alternatively as the loss of a *C-*pentosyl fragment and water (142 Da = 88 + 3 × 18) in combination with a *C*-deoxyhexosyl fragment (120 Da). Based on MS data alone, it was not possible to unambiguously determine which glycosyl residue occupied the C6 or C8 position; therefore, both 8-*C*-(deoxyhexosyl)-6-*C*-pentosyldelphinidin and 6-*C*-(deoxyhexosyl)-8-*C*-pentosyldelphinidin remain plausible suggestions.

Compound 7 is hypothesized to exist predominantly in the chalcone form. This interpretation is supported by the deduced neutral aglycon mass (320 Da). This is consistent with delphinidin (303 Da) followed by the addition of water (+18 Da), and deprotonation (−1 Da) in negative ion mode or protonation (+1 Da) in positive ion mode. Furthermore, the UV–Vis spectrum of the eluted sample that corresponds to this peak exhibits a major absorption at ∼280 nm and ∼330 nm, attributed to the chalcone form of an anthocyanin, and a minor absorption at approximately 520 nm which may be attributed to other coeluting pigments. Due to the relatively slow rate of equilibration between chalcone and flavylium forms, they can elute as distinct peaks.^43,44^

At 1% (v/v) formic acid in methanol in which the pigments were dissolved prior to chromatographic separation, a pH of ∼2 enables equilibration of the flavylium cation to the colorless carbinol pseudobase,^45,46^ which can subsequently tautomerize to the chalcone form.^47^ Notably, 6,8-di-*C*-glycosylated anthocyanidins have been reported to exist predominantly in the chalcone form at pH 4.5^48^ and a significant fraction may persist in this form even at lower pH owing to stabilization by intramolecular hydrogen bonding. Specifically, hydrogen bonding between the 2‴–OH group of the C8 glycoside and the phenolic OH at C7 of ring A can possibly stabilize the chalcone structure.^49^ Equilibration of the flavylium cation of 6,8-di-*C*-glycosylated anthocyanidins to its carbinol form in the sample solution followed by hydration and tautomerization processes can therefore account for the chalcone formation and the corresponding peak at 25.1 min.

In contrast to *C*-glycosylation, *O*-glycosylation of proanthocyanidins has been more widely reported. Examples include 3-*O*-xylosides,^24^ 3-*O-*rhamnoside,^22^ 3-*O-*glucoside,^23^ and *O-*polygalacturonate conjugates,^25^ which could be well present also in WRC bark extracts. However, the acid lability of *O-*glycosidic linkages accounted for their cleavage upon acidic butanol hydrolysis and thus prevented their detection. By contrast, flavonoid *C*-glycosides are considerably more resistant to acidic hydrolysis, rendering them readily observable under these conditions.^50^ On the one hand, presence and extent of proanthocyanidin *O*-glycosides in WRC bark remained unresolved, on the other hand, the used protocol, which effectively “blinded out” those *O*-glycosides by hydrolysis, thus enabled easy *C*-glycoside detection, which would have been extremely challenging – if not outright impossible – in the presence of a large excess of *O*-glycosides.

#### 3.1.2 NMR Analysis of the Depolymerized WRC Bark AIR

In addition to chromatography and MS data (Figures 1 and 3), NMR spectroscopy (Figures 4 and 5; Figures S5 and S6) was employed to obtain complementary information on *C*-glycosyl-anthocyanidin structures. Several correlations were identified that are consistent with the proposed structures based on LC–MS. Together, these are used to infer the linkage in the proposed flavoglycan structural model. In the following, where appropriate, tentative assignments are given and discussed in the context of published chemical shifts of anthocyanins.

The ^1^H NMR spectra (Figure 6A) display resonances highly downfield at approximately 8.6 ppm, which are attributed to the C4–H proton of anthocyanidin ring C.^51^ Signals in the range of 6.5–8.0 ppm (Figure 6A, inset A1) are consistent with aromatic protons of rings A and B of anthocyanidins.^51^ However, assignment within this region is compromised by overlap with the solvent peak arising from the exchangeable proton of trifluoroacetic acid, which obscures resonances between approximately 6.5 and 7.5 ppm. Resonances between 3.0 and 5.0 ppm were assigned to carbohydrate-derived protons (Figure 6A, inset A2). Intense signals below 3 ppm indicate the presence of aliphatic moieties, likely arising from butyl substituents formed during acidic butanol hydrolysis and/or residual fatty acids.

**Figure 6:**
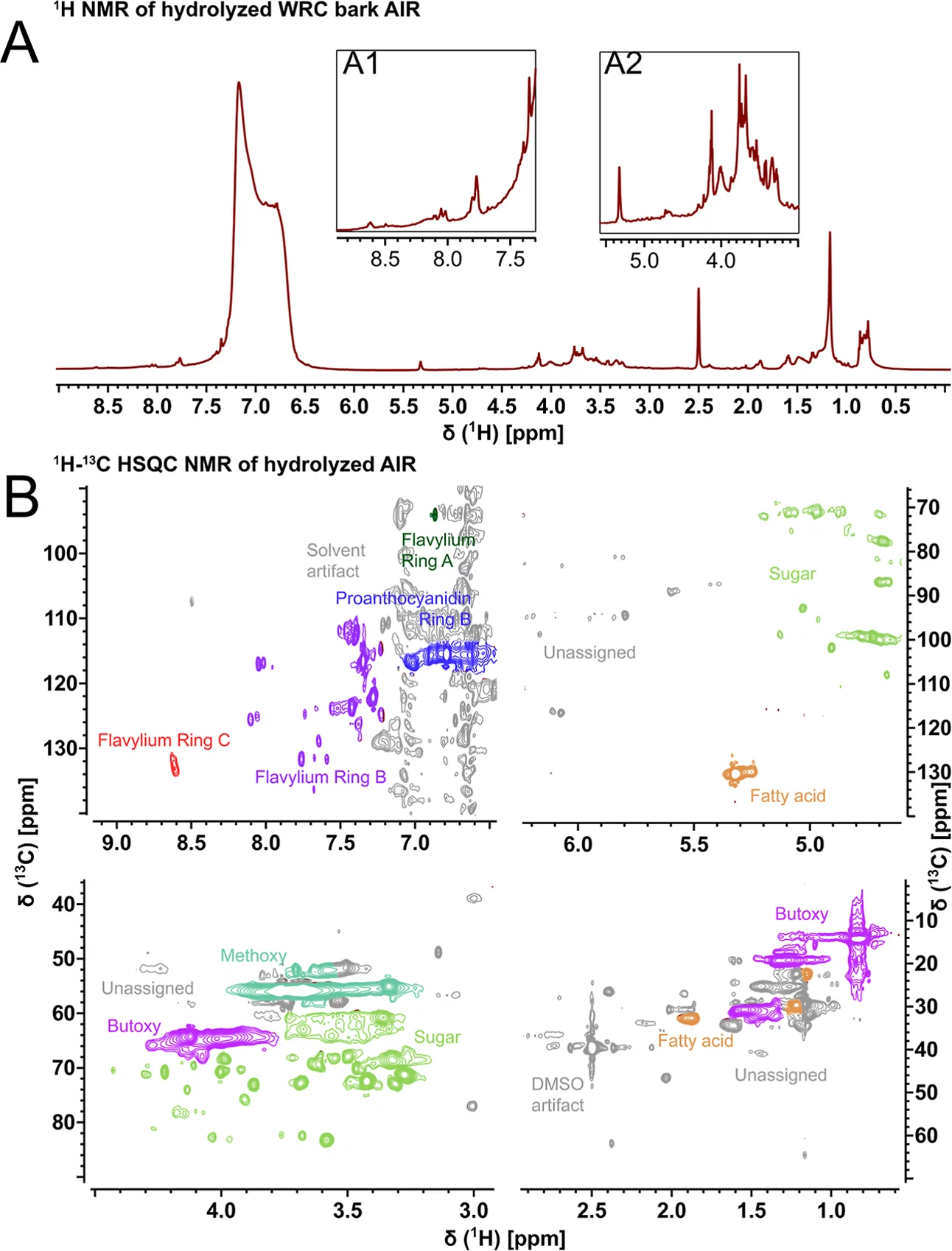
^1^H NMR spectra (A) and ^1^H–^13^C HSQC NMR spectra (B) of depolymerized WRC bark AIR.

The HSQC spectra (Figure B) reveal cross-peaks corresponding to aromatic protons of rings B and C of flavylium compounds, consistent with anthocyanidin structures.^51^ In contrast, signals attributable to ring A protons (C6–H and C8–H) were difficult to identify unambiguously due to interference from solvent-related resonances. A cross-peak at δ_H_ 6.86 ppm/δ_C_ 94.02 ppm is tentatively assigned to an anthocyanidin C6–H proton, supported by HMBC correlations discussed below.

Prominent cross-peaks at δ_H_ 6.50–7.00/δ_C_ 115–117 ppm are characteristic of aromatic protons from procyanidin ring B (CH-2′, CH-5′, and CH-6′).^52^ Consistent with this interpretation, LC–MS chromatograms of the depolymerized and purified sample still showed a broad peak absorbing at 280 nm, indicative of residual proanthocyanidins (Figure S10).

Downfield aromatic cross-peaks at δ_H_ 8.05/δ_C_ 117.07 ppm; δ_H_ 8.10/δ_C_ 125.66 ppm are tentatively assigned to CH-2′/CH-5′ and CH-6′ protons of flavylium ring B, predominantly from cyanidin units.^51^ Additional signals clustering at δ_H_ 7.30–7.45/δ_C_ 112–132 ppm likely arise from ring B protons of other flavylium derivatives, such as delphinidin and peonidin.^51^

Methoxyl groups (−O–CH_3_) were identified by cross-peaks at approximately δ_H_ 3.70/δ_C_ 55.72 ppm,^46^ while the butoxyl groups’ methylenoxy (−O–CH_2_−) was assigned to δ_H_ 4.12/δ_C_ 64.46 ppm, supported by HMBC correlations (Figure 7C). The additional resonances corresponding to the butyl chain were observed upfield at δ_H_ 0–1.5/δ_C_ 10–30 ppm. Numerous carbohydrate-related signals were also detected in the oxygenated aliphatic region, including anomeric CH signals at δ_H_ 4.50–5.25/δ_C_ 95–105 ppm.

**Figure 7:**
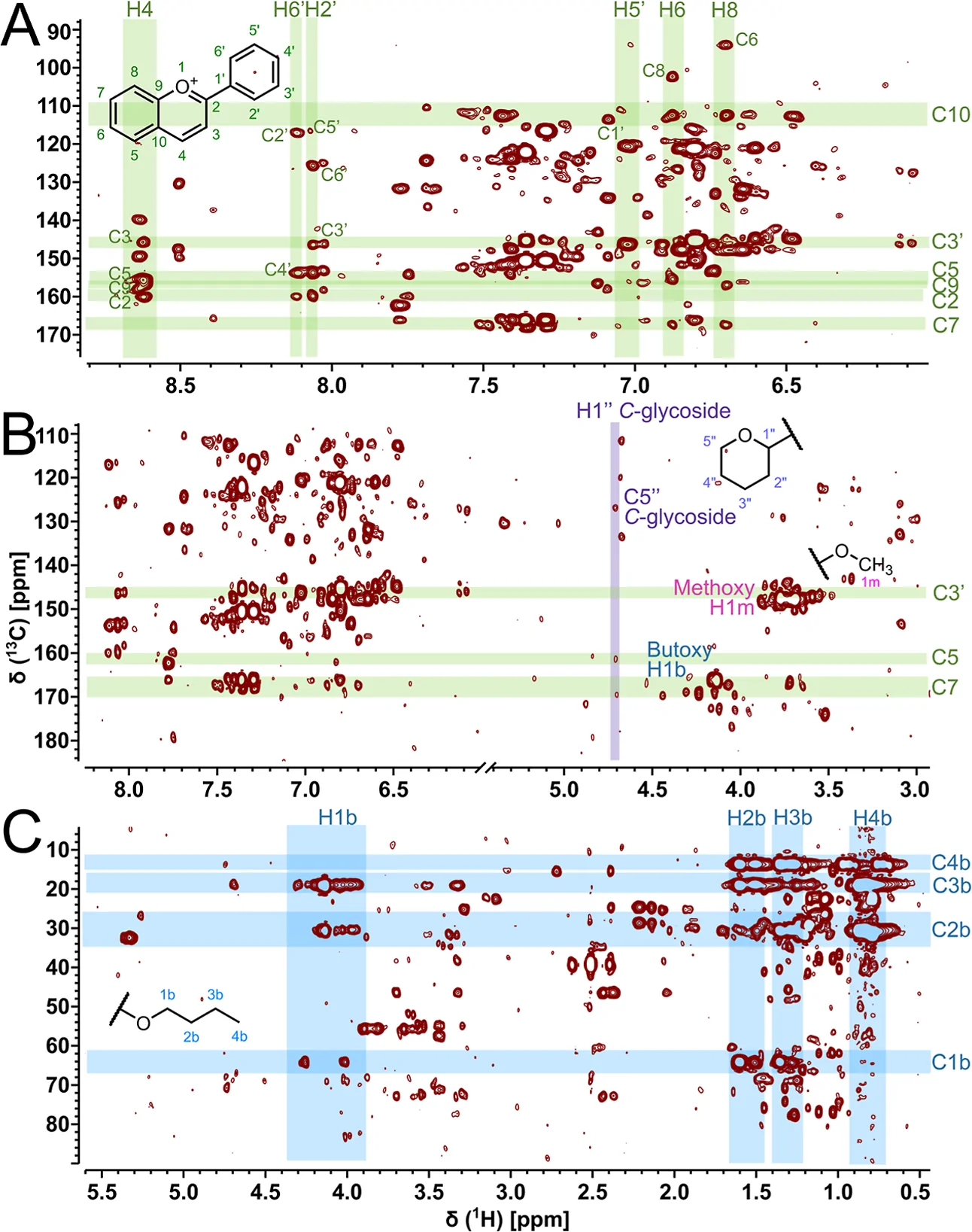
^1^H–^13^C HMBC NMR spectra of depolymerized WRC bark AIR. Correlations in the flavylium structure (A); Methoxylation, butoxylation and *C*-glycosylation (B); and butyl chain (C). Green labels refer to numbering in the flavylium structure, purple labels refer to sugar residue, pink label refers to methoxy group, and blue labels refer to butoxy group.

The corresponding HMBC spectrum (Figure 7; Figure S6) provided further support for the presence of anthocyanidin structures. Correlations corresponding to aromatic protons of a cyanidin nucleus are evident (Figure 7A) and the key correlations are summarized in Figure S9A and Table S5. Given that catechin units constitute the dominant building blocks of proanthocyanidins in WRC bark AIR,^28^ cyanidin is expected to be the most abundant anthocyanidin formed upon depolymerization. This was found consistent with the LC–MS results. Accordingly, intense long-range correlations involving resonances near δ_H_∼8.6 ppm were tentatively assigned to cyanidin-derived flavylium protons, enabling partial reconstruction of the flavylium framework.

In Figure 7B, correlation at δ_H_ 3.70/δ_C_ 147 ppm is consistent with a methoxyl group attached to flavylium ring B, most plausibly at C3′. This supports the LC–MS-based identification of peonidin. Conversely, the correlation at δ_H_ 4.12/δ_C_ 166 ppm suggests a butoxyl group attached via an ether linkage to an aromatic system. Possible attachment sites include phenolic oxygens at C5 or C7 of ring A or at C4′ of ring B.

The relatively weak intensity of carbohydrate-derived signals in the ^1^H and HSQC spectra, compared to those observed in native WRC bark AIR,^28^ indicates that most polysaccharides were removed during post-hydrolytic solid-phase extraction. Nonetheless, residual carbohydrate-related signals remain detectable in the HSQC and HMBC spectra (Figures 4B and 5B), suggesting the presence of a minor fraction of acid-resistant glycosidic linkages. Concurrent enrichment of phenolic signals further supports selective retention of anthocyanidin-derived species in the NMR sample.

Cross-peaks at δ_H_ 4.5–5/δ_C_ 70–85 ppm observed in the HSQC spectrum (Figure 6B) are consistent with anomeric protons vicinal to aromatic carbons connected by a C–C bond, characteristic of flavonoid *C*-glycosides.^48,53^ Note that the anomeric protons in such aromatic *C*-glycosides formally become benzylic (δ_H_ ∼4.5).

Additional HMBC correlations at δ_H_ 4.7/δ_C_ ∼161 and ∼170 ppm may arise from long-range coupling between carbohydrate anomeric protons (H-1″) and carbons C5 and C7 of flavylium ring A, respectively.^47^ Furthermore, a correlation at δ_H_ 4.70/δ_C_ 68.75 (Figure 6C) ppm reflects coupling between the anomeric proton and a carbohydrate ring carbon (e.g., C5″ of xylose or rhamnose).^51^ These key correlations are summarized in Figure S9B.

Several cross-peaks corresponding to potential anomeric CH groups were also observed at δ_H_ 4.7–5/δ_C_ ∼100 ppm in the HSQC spectrum. Inspection of the HMBC spectrum in this region did not reveal the expected correlations to pyranose ring carbons or aromatic carbons, making assignments as *O*-glycosylated anthocyanidins unlikely. This outcome is consistent with the known acid lability of *O*-glycosidic linkages under acidic butanol hydrolysis conditions. Nevertheless, this evidently does not exclude the occurrence of proanthocyanidin *O*-glycosides in native WRC bark. It just points to the fact that milder depolymerization strategies would be required to preserve and detect such linkages.

Additional HSQC–TOCSY and COSY experiments (Figures S7 and S8) did not enable further resolution of carbohydrate resonances. Taken together, the NMR data obtained here cannot unequivocally define the exact position of *C*-glycosylation in the anthocyanidin products formed during acidic butanol hydrolysis. However, the observed correlations are consistent with the presence of low-abundance, acid-stable *C*-glycosylated anthocyanidins which were inferred from LC–MS analysis.

### 3.2 Proposed Structure of WRC Bark Flavoglycans

Based on the mass spectrometric and NMR evidence in this study, we propose a tentative flavoglycan structural model depicted in Figure 8. In this proposed model, *C-*glycosylation between proanthocyanidins and polysaccharides leads to the formation of flavoglycan structures as hypothesized in WRC bark AIR. The three distinct *C*-glycosylanthocyanidins (Figure 2) are the presumed interpolymer linkage residues in flavoglycans in the proposed structural model in Figure 8. In the proposed structural model, polysaccharides of varying chain lengths are covalently linked to proanthocyanidins through acid-stable *C*-glycosidic bonds. Owing to the inherent heterogeneity of both the polysaccharide and proanthocyanidin components, flavoglycans are expected to exist as a structurally diverse population of glycoconjugates spanning a broad range of molecular weights, chain lengths, compositions, and *C*-glycosylation patterns.

**Figure 8:**
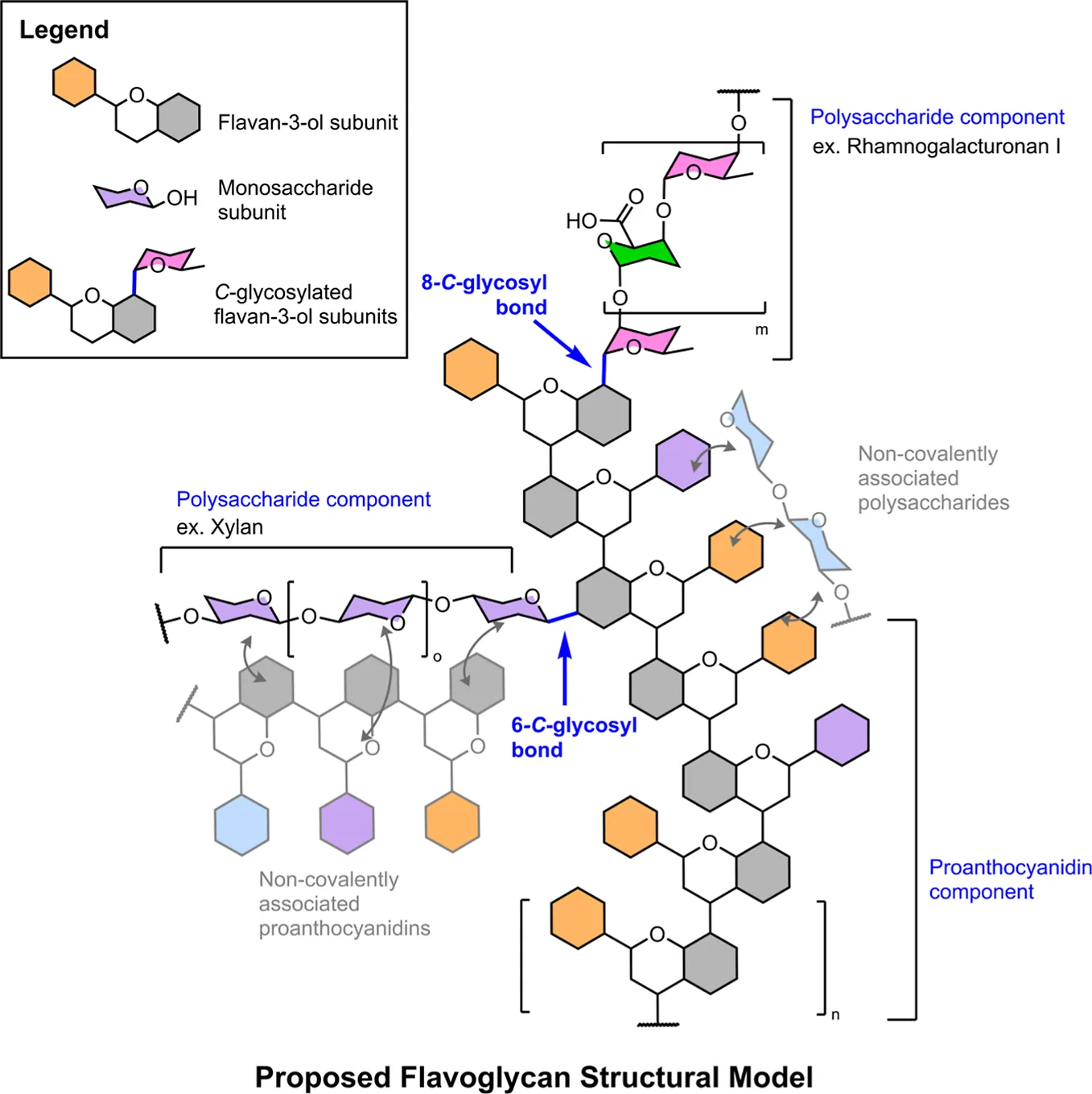
Tentative structural model of flavoglycans from WRC bark AIR. Polysaccharides and proanthocyanidin chains can exist in varying mean degrees of polymerizations *n*, *m*, *o*. The flavoglycans are expected to exist in a broad distribution of chain lengths of the corresponding macromolecule components.

The detection of such flavoglycans in the aqueous extract of WRC bark provides a conceptual framework for understanding the persistent association between proanthocyanidins and polysaccharides in bark-derived extracts. More broadly, it establishes a foundation for future investigations aimed at elucidating the native structure, biosynthetic origin, and functional role of flavoglycans, as well as exploring their potential valorization as unique polyphenol–carbohydrate hybrid materials.

Previous reports have only been able to show the participation of glucose (a hexose) in the *C*-glycosylation of proanthocyanidins. For example, 6-*C*- and 8-*C*-glucosylated procyanidin dimers have been isolated from cinnamon bark.^18^ In adzuki beans, only 6-*C*-glucosylated epicatechin extension units was isolated.^26^ The present work complements these findings by providing supporting evidence for the involvement of pentoses and deoxyhexoses. The detection of *C-*glycosylanthocyanidins bearing such carbohydrate residues suggests participation of hemicelluloses (*e.g.*, xylose from xylans, arabinose from arabinans) and pectins (*e.g*., rhamnose from rhamnogalacturonan I) in the covalent attachment of proanthocyanidins to those cell wall polysaccharides. This informs the range of possible carbohydrates in the proposed flavoglycan structural model (Figure 8).

Based on thiolysis results, WRC bark AIR proanthocyanidins are primarily (+)-catechin-based.^28^ Since catechin units exhibit strong affinities to polysaccharides,^54^ the extent of proanthocyanidin binding and accumulation in WRC cell walls is expected to be significant. The conformational compatibility of methyl-esterified pectins, acetylated xylans, and xyloglucans likely promotes strong associations with catechin-based proanthocyanidins.^3,28,55^ Such spatial proximity may favor endogenous crosslinking reactions between the two macromolecules. The detection of peonidin following acidic butanol hydrolysis further suggests methylation of certain procyanidin units. This is a modification commonly observed with flavonoids deposited in the cell wall.^40^ Thus, proanthocyanidins may become integral components of the plant cell wall through covalent linkages in addition to the well-documented non-covalent interactions.^3–5,28^

The findings of this study complement other known modes of polyphenol-mediated cell wall crosslinking, including diferulate and *p-*coumaric acid crosslinks with pectins,^56,57^ isodityrosine crosslinks in glycoproteins,^58,59^ and the diverse covalent linkages reported between lignin and polysaccharides.^60,61^ Continued progress in polysaccharide and proanthocyanidin analytical methodologies will likely uncover additional types of carbohydrate–polyphenol linkages, ultimately providing a more complete understanding of the structural organization, properties, and biological roles of flavoglycans in plant cell walls.

The quantities of compounds 1, 2, and 7 were estimated as cyanidin equivalents, yielding values of 0.02, 0.7, and 0.1 nmol cyanidin equivalents per gram of AIR, respectively. In total, approximately 0.8 nmol of interpolymer linkages per gram of AIR were detected. This corresponds to roughly 64 μmol of interpolymer linkages per 100 mol of monosaccharide units in AIR, or about 2 mmol of interpolymer linkages per 100 mol of flavan-3-ol units. While there is no evidence that more than one polysaccharide chain is attached to a proanthocyanidin chain, this is not deemed impossible. Considering that the yield of anthocyanidin from proanthocyanidin under optimum conditions was reported only to be about 58% mol,^29^ the quantities reported here are at a best a lower limit estimate. These values should be regarded as semi-quantitative estimates intended to provide an order-of-magnitude assessment of the frequency of this type of covalent crosslinking.

Even at these low estimated levels of occurrence, such covalent linkages are expected to exert a measurable influence on the physicochemical properties of flavoglycans. These effects may include changes in hydration, solubility, hydrodynamic size, solution viscosity, chemical reactivity, and resistance to depolymerization.^62^ Owing to the substantially higher hydrophilicity of polysaccharides relative to proanthocyanidins, even a single covalently attached carbohydrate chain may significantly alter the molecular interactions and interfacial behavior of the resulting glycoconjugate in aqueous environments. Similar effects have been observed in glycoproteins, where the introduction of only one or a few glycosylation sites can profoundly modify solution and interfacial properties.^62^

Although the precise properties and biological functions of flavoglycan structures remain incompletely understood, the presence of *C*-glycosylation in the structure possibly confers enhanced resistance to acidic and enzymatic hydrolysis compared with *O*-glycosylation.^63^ This may explain the persistence and unusual behavior of proanthocyanidin–polysaccharide complexes reported in earlier studies.^14,15,64^ These characteristics may be further influenced by the Wessely-Moser rearrangement of *C*-glycosylated flavonoids^65^ and by the propensity of depolymerization products to undergo secondary condensation reactions. These features make flavoglycans advantageous as functional food components expected to remain relatively stable under harsh conditions (heat, pH, and oxidation). Polyphenol-rich carbohydrates may show improved swelling capacity in oil and/or water, as well as water holding effects.^66^ This slows down their absorption, making these dietary fibers available for utilization by the microbiome in the colon.^67^ While bare flavan-3-ols are bitter, rough, and puckering astringent, *C*-glycosylated flavan-3-ols provide a smooth velvety mouthfeel with no bitterness.^68^ It is possible that these organoleptic properties also apply to flavoglycans, a subject that deserves further studies. While the laboratory synthesis of flavan-3-ols *C*-glycosylated with oligo- and polysaccharides has already been reported by Stark et al.,^69^ there is currently no known synthesis protocol for flavoglycans as represented in the proposed structural model shown in Figure 8.

### 3.3 Limitations and Challenges in the Analysis of Flavoglycans

Definitive structural elucidation of flavoglycans remains challenging owing to the intrinsic complexity of botanical extracts, the trace abundance of the target species, the absence of established methods for isolating intact flavoglycan macromolecules, and the current limitations in instrumental sensitivity. The following discussion outlines how these factors influence the interpretation of the present results and the proposed structural model (Figure 8).

*C*-glycosylation is generally regarded as a rare type of linkage that occurs only at trace levels in plant tissues. For example, only approximately 1 ppm of *C*-glucosylated procyanidin dimers has been reported in cinnamon bark.^18^ Likewise, approximately 1.5 ppm of 6-*C*-glucosylated epicatechin extension units has been detected in adzuki beans.^26^ Similar to those studies, the present work required large-scale biomass processing (>50 kg) and extensive purification through multiple preparative chromatographic steps to isolate only milligram quantities of *C*-monoglycosylated proanthocyanidins suitable for structural characterization.

These analytical challenges are further compounded by the relatively low recovery of anthocyanidins and *C*-glycosylated anthocyanidins obtained under the mild acidic butanol hydrolysis conditions employed in this study. Residual proanthocyanidins remain in the hydrolysate and complicate subsequent analyses. This behavior may result from the formation of proanthocyanidin–anthocyanidin complexes,^70^ which are difficult to separate even using preparative chromatography and solid-phase extractions.

The analysis is further complicated by the presence of oxidized proanthocyanidins in aged plant tissues such as bark, which are known to resist acidic depolymerization.^71^ It has been estimated that more than one-third of proanthocyanidins in such tissues remain inaccessible to structural characterization using conventional depolymerization methods. Regardless of the depolymerization strategy employed, WRC bark AIR consistently yielded both recalcitrant fractions and highly reactive fractions that underwent secondary condensation, resulting in the formation of new polymeric products and only partial recovery of the target species.

Conversely, extending the duration of acidic butanol hydrolysis introduces additional side reactions, including rearrangement, degradation, polymerization, and formation of anthocyanidin–butanol adducts. These side products further increase the complexity of both the LC–MS chromatograms and the NMR spectra. A potential concern is whether *O*-glycosides originally present in WRC bark AIR could rearrange to *C*-glycosides during hydrolysis.^72^ However, such rearrangements generally require appropriate Lewis acid catalysts. Under the strongly acidic conditions employed in this study, the predominant reaction pathway is expected to be cleavage of *O*-glycosidic bonds with formation of the corresponding aglycons, rather than *O*→*C*-glycoside rearrangement.^39^ In addition, polymeric pigments generated during hydrolysis produce broad unresolved chromatographic signals that partially overlap with monomeric anthocyanins. Owing to their structural similarity, these polymeric species also exhibit comparable chromatographic retention, UV–Vis absorption profiles, MS fragmentation behavior, and NMR chemical shifts, further complicating structural assignments.

Natural structural heterogeneity further complicates the detection and quantification of flavoglycans. Unlike monoglycosylated proanthocyanidins, flavoglycans comprise polymeric carbohydrates covalently linked to polymeric proanthocyanidins, resulting in numerous possible combinations of carbohydrate residues, polyphenolic units, and chain lengths. This structural diversity presents a substantially greater analytical challenge for degradative approaches.^64,73^ Consequently, the abundance of interpolymer covalent linkages reported in this study is likely underestimated.

The compositional complexity of the depolymerized samples also limits unequivocal structural assignment by NMR spectroscopy alone. The coexistence of multiple anthocyanidin derivatives, residual proanthocyanidins, and diverse carbohydrate moieties results in extensive spectral overlap. Some correlations may have remained undetected because of the extremely low abundance of *C-*glycosylated anthocyanidins. Furthermore, the absence of additional corroborating HMBC and HSQC correlations within the carbohydrate residues or to additional flavylium carbons limits the confidence of individual NMR assignments, which should therefore be regarded as tentative. Additional uncertainty arises from the lack of authentic analytical standards for *C-*glycosylated flavan-3-ols and anthocyanins, which would greatly facilitate the identification and quantification of these compounds in complex botanical matrices. For these reasons, the proposed structures are interpreted from the combined evidence provided by LC–MS and NMR rather than from either technique alone.

Despite these limitations, the results presented here provide converging lines of experimental evidence supporting the existence of covalently associated proanthocyanidins and polysaccharides in WRC bark. Overall, the available data are most consistent with a structural model in which these two macromolecular components are linked through *C-*glycosidic bonds. Although alternative interpretations cannot be completely excluded, a purely non-covalent association does not adequately explain the observed resistance to acidic depolymerization together with the inability to separate the two components using multiple inert solvent systems. Nevertheless, the proposed flavoglycan model should be regarded as tentative, and additional orthogonal analytical approaches will be required to establish the molecular structure of flavoglycans unambiguously.

In this study, we investigated whether proanthocyanidins and cell wall polysaccharides in western redcedar (WRC) bark are covalently linked through *C-*glycosylation. The combined LC–MS and NMR analyses of acidic butanol hydrolysis products revealed the presence of *C-*glycosylated anthocyanidins, providing experimental evidence consistent with the occurrence of *C*-glycosylated proanthocyanidins in WRC bark AIR. Based on these findings, we propose a tentative structural model for flavoglycans in which proanthocyanidins and cell wall polysaccharides are associated through a combination of *C-*glycosidic linkages and non-covalent interactions.

From a practical perspective, these findings suggest that strategies for the valorization of WRC bark should consider not only freely extractable or non-covalently associated proanthocyanidins and polysaccharides, but also their covalently linked counterparts. The water extractability of flavoglycans may facilitate their incorporation into food systems, consistent with traditional uses of WRC bark as teas or flavoring agents. Furthermore, a better understanding of the structure and physicochemical properties of flavoglycans may enable the development of new applications that exploit their anticipated stability, interfacial behavior, and texture-modifying capabilities. Beyond food and beverage systems, these unique polyphenol–carbohydrate conjugates may also offer opportunities for future applications in agriculture, nutrition, health, and other bio-based technologies.

## Supplementary Material



## Notes

Additional experimental details, materials and methods, and information on proanthocyanidin and carbohydrate composition of WRC bark AIR, the acidic butanol control experiments, anthocyanidin yield after acidic butanol hydrolysis, full NMR spectra of depolymerized WRC bark AIR, and possible fragmentation pathways supporting LC-MS results (PDF)

## Abbreviations Used

AIR: alcohol-insoluble residue
COSY: ^1^H–^1^H correlation spectroscopy
HMBC: ^1^H–^13^C heteronuclear multiple bonding correlation
HSQC: heteronuclear single quantum coherence
HSQC-TOCSY: heteronuclear single quantum coherence total correlation spectroscopy
LC–MS: liquid chromatography–mass spectrometry
mDP: mean degree of polymerization
NMR: nuclear magnetic resonance
PC/PD: procyanidin-to-prodelphinidin ratio
PVPP: polyvinylpolypyrrolidone
TFA: trifluoroacetic acid
WRC: western redcedar
