# Supplementary material for: Flavoglycans: Evidence for Covalent Proanthocyanidin–Polysaccharide Linkages in Western Redcedar Bark
Source: J Agric Food Chem. 2026 Sep 10;74(37):29020–32. doi: 10.1021/acs.jafc.6c03146 (PMC13614299; doi:10.1021/acs.jafc.6c03146)
Supplement: Supplementary file 1 [file jf6c03146_si_001.pdf]

Supporting Information

Flavoglycans: Evidence for Covalent Proanthocyanidin-Polysaccharide Linkages in Western Redcedar Bark

Gio Ferson M. Bautista[a,b], Oliver Musl[c,], Markus Bacher[c], Zhicheng Xia[a], Julia L. Azzi[b,d], Antje Potthast[c], Thomas Rosenau[c], Orlando J. Rojas[a,b,d,e,*]*

[a] Department of Chemistry, The University of British Columbia, 2036 Main Mall, Vancouver, BC V6T 1Z1, Canada

[b] Bioproducts Institute, University of British Columbia, 2385 East Mall, Vancouver, BC V6T 1Z4, Canada

[c] Department of Natural Sciences and Sustainable Resources, Institute of Chemistry of Renewable Resources, BOKU University, Konrad-Lorenz-Strasse 24, 3430 Tulln, Austria

[d] Department of Chemical and Biological Engineering, University of British Columbia, 2360 East Mall, Vancouver, BC V6T 1Z3, Canada

[e] Department of Wood Science, The University of British Columbia, 2424 Main Mall #2900, Vancouver, BC V6T 1Z1, Canada

*Corresponding authors: O.M.: oliver.musl@boku.ac.at; O.J.R.: orlando.rojas@ubc.ca

Table of Contents

Section		Page
**I**	Supplementary methods	**2**
**II**	Supplementary tables	**4**
**III**	Supplementary figures	**9**
**IV**	Fragmentation reaction schemes	**17**

I. Supplementary Methods

Determination of acidic butanol hydrolysis products by HPLC-DAD and HPLC-DAD-MS. Products of acidic butanol hydrolyses were analyzed in an Agilent HPLC 1260 equipped with a diode array detector and a fluorescent light detector. The column used was a Poroshell HPH-C18 2.7 µm (4.6 x 100 mm). A flow rate of 0.6 mL/min at 30 °C was used with Solvent A as 0.13% (v/v) TFA in water and Solvent B as 0.10% (v/v) TFA in acetonitrile. The solvent programming was as follows: 0-10.0 min 5% B, 10.0-15.0 min increase to 20% B, 15.0-20.0 min 20% B, 20.0-25.0 min increase to 55% B, 25.0-32.0 min 55% B, 32.0-33.0 min increase to 95% B, 33.0-38.0 min 95% B, 38.0-39.0 min decrease to 5% B, 39.0-44.0 min 5% B. The sample mixture was filtered through a centrifugal spin filter with a 0.22 µm cellulose acetate membrane prior to the injection of 5 µL of the sample. The absorbance of the eluate at 190-950 nm was monitored.

For the ESI-Ion Trap-MS^n analysis, a Hewlett Packard HPLC 1100 equipped with a diode array detector and a Bruker HCT Ultra PTM Discovery System was used. The column used was Atlantis T3 3 µm (3.0 x 100 mm). A flow rate of 0.5 mL/min at 30 °C was used with Solvent A as 0.13% (v/v) formic acid in water and Solvent B as 0.10% (v/v) formic acid in acetonitrile. The solvent programming was as follows: 0-13.0 min 5% B, 13.0-18.0 min increase to 20% B, 18.0-23.0 min 20% B, 23.0-28.0 min increase to 55% B, 28.0-35.0 min 55% B, 35.0-36.0 min increase to 95% B, 36.0-41.0 min 95% B, 41.0-42.0 min decrease to 5% B, 42.0-47.0 min 5% B. The sample

mixture was filtered through a centrifugal spin filter with a 0.22 μm cellulose acetate membrane prior to the injection of 5 μL of the sample. The absorbance of the eluate at 190-950 nm was monitored. The ESI-MS analysis was done in negative ion mode and positive ion mode at an m/z range of 150-1500 m/z, and an average of 5 spectra were collected for each data point. In positive ion mode, capillary voltage of -5000 V, nebulizer pressure of 70 psi, and dry gas flow rate of 10 L/min at 365 °C were used. The Smart Setting of the software was set to a target mass of 500 m/z, a compound stability of 50%, and trap drive level of 80%. At these settings, the following were automatically set by the software: skimmer of 40.0 V, capillary exit of 109.8 V, Oct 1 DC of 8 V, Oct 2 DC of 1.7 V, Octopole RF of 187.1 Vpp, Lens 1 of -5 V, Lens 2 of -60 V, and a trap drive of 48.3. ICC was activated and manually set to a value of 100,000 and a maximum accumulation time of 100 ms. In negative ion mode, capillary voltage of +3500 V, nebulizer pressure of 60 psi, and dry gas flow rate of 11 L/min at 330 °C were used. The Smart Setting of the software was set to a target mass of 500 m/z, a compound stability of 80%, and a trap drive level of 120%. At these settings, the following were automatically set by the software: skimmer of 40.0 V, capillary exit of -121 V, Oct 1 DC of- 8 V, Oct 2 DC of -1.7 V, Octopole RF of 187.1 Vpp, Lens 1 of 5 V, Lens 2 of 60 V, and a trap drive of 63.7. ICC was activated and manually set to a value of 10,000 and a maximum accumulation time of 100 ms. For MS^n fragmentations in positive and negative ion mode, an isolation width of 4 m/z was used, and the fragmentation amplitude was adjusted from 0.5-2.0 V.

II. Supplementary Tables

Table S1. Monosaccharide content of dried WRC bark, WRC bark after extraction, and AIR.

Material	Mass of monosaccharide ± SD (n=3) WRC bark (%w/w in unextracted dried bark)	WRC bark after extraction (%w/w in unextracted dried bark)	AIR (%w/w in AIR)
**Total hydrolysis in H_2SO_4[a]**	43 ± 4	43 ± 7	18 ± 2
Glu	30 ± 3	31 ± 5	9 ± 1
Ara	1.14 ± 0.08	0.9 ± 0.2	1.9 ± 0.4
Gal	1.2 ± 0.1	1.0 ± 0.3	2.1 ± 0.5
Xyl	4.1 ± 0.5	3.9 ± 0.6	2.28 ± 0.07
Man	6.2 ± 0.5	6.5 ± 0.9	2.7 ± 0.1
Rha	0 (not detected)	0 (not detected)	0 (not detected)
Fuc	1.03 ± 0.03	0.7 ± 0.2	1.47 ± 0.07
Acid-insoluble residue after total hydrolysis[b]	62 ± 8	51 ± 4	90 ± 10
**Acidic methanolysis[c]**	14.5 ± 0.5	13.7 ± 0.4	21.9 ± 0.6
Glu	1.37 ± 0.06	1.30 ± 0.04	2.9 ± 0.1
Ara	1.31 ± 0.06	1.15 ± 0.05	2.4 ± 0.1
Gal	1.16 ± 0.09	1.07 ± 0.05	2.5 ± 0.8
Xyl	2.7 ± 0.01	2.59 ± 0.09	1.72 ± 0.07
Man	3.5 ± 0.3	3.5 ± 0.2	1.85 ± 0.06
Rha	0.34 ± 0.04	0.29 ± 0.03	1.33 ± 0.04
Fuc	0.21 ± 0.04	0.19 ± 0.02	0.40 ± 0.02
GalA	2.3 ± 0.3	2.0 ± 0.2	7.8 ± 0.3
4-*O*-MeGlcA	1.64 ± 0.09	1.6 ± 0.1	0.59 ± 0.02
GlcA	0 (not detected)	0 (not detected)	0.41 ± 0.02
**Driselase digestion (% w/w)[a]**	n/a[d]	n/a[d]	11.3 ± 0.9
Glu			1.3 ± 0.3
-Glu from isoprimeverose			0.34 ± 0.03
-Glu as a monosaccharide			1.0 ± 0.3
Ara			0.80 ± 0.06
Gal			0.84 ± 0.06
Xyl			1.06 ± 0.05
-Xyl from xylobiose			0.55 ± 0.04
-Xyl from isoprimeverose			0.28 ± 0.02
-Xyl as a monosaccharide			0.228 ± 0.009
Man			0.238 ± 0.001
Rha			0.338 ± 0.008
Fuc			0.56 ± 0.07
GalA			6.1 ± 0.9
4-*O*-MeGlcA			0 (not detected)
GlcA			0 (not detected)

[a]Quantified by HPAEC–PAD; [b]Dry sample basis; [c]Quantified by GC–MS; [d]Not analyzed.
Abbreviations are defined as follows: Glu- glucose; Ara- arabinose; Gal- galactose; Xyl- xylose;

Man- mannose; Rha- rhamnose; Fuc- fucose; GalA- galacturonic acid; 4-*O*-MeGlcA- 4-*O*-methylglucuronic acid; GlcA- glucuronic acid

Table S2. Retention time and mass spectra (MS) information for proanthocyanidin terminal and extension units after thiolysis.

Peak #	Compound	Retention time, min	MS^n Precursor and fragment ions (−ve), m/z	Amount ± SD (% w/w)[a] (n=3)
1	(+)-gallocatechin	2.0	419.0 → 304.9, 288.9	0.054 ± 0.004
2	(−)-epigallocatechin	2.5	419.0 → 304.9, 288.9	0.008 ± 0.001
3	(+)-catechin	3.2	403.0, 289.0	0.121 ± 0.005
4	(−)-epicatechin	4.5	403.0, 289.0	0.095 ± 0.005
5	3,4-*trans*-gallocatechin benzyl thioether	10.4	541.2 → 427.0, 302.9	0.072 ± 0.008
6	3,4-*cis*-gallocatechin benzyl thioether	10.7	541.2 → 481.1, 427.0, 303.1, 177.0	0.045 ± 0.002
7	3,4-*trans*-epigallocatechin benzyl thioether	11.2	541.2 → 416.9, 302.9	0.139 ± 0.05
8	3,4-*trans*-catechin benzyl thioether	13.0	525.2 → 400.9, 286.7	0.119 ± 0.002
9	3,4-*cis*-catechin benzyl thioether	14.2	525.2 → 410.9 → 286.9, 243.0, 125.0	0.70 ± 0.02
10	3,4-*trans*-epicatechin benzyl thioether	15.6	525.3 → 401.1, 287.0	0.110 ± 0.003
Total proanthocyanidin yield (% w/w)[a]			1.46 ± 0.04	
Number average DP			5.3 ± 0.2	
Number average MW, kDa			1.32 ± 0.05	
PC:PD ratio			3.90 ± 0.83	
cis:*trans* ratio			0.40 ± 0.04	

[a]Based on the dry weight of AIR

Table S3. Acidic butanol hydrolysis of mixtures of procyanidins and polysaccharides.

	Flavanol	Sugar	Monomers m/z (−ve)*		Adducts m/z (−ve)*			
			285	289	417 Pentose (531)	431 Deoxyhex (545)	447 Hexose (561)	461 Hexuronic (575)
**1**	Procyanidin B3	-	✓	X	X	X	X	X
**2**		Pectin	✓	X	X	X	X	X
**3**		Xyloglucan	✓	X	X	X	X	X
**4**		Xylan	✓	X	X	X	X	X
**5**		Starch	✓	X	X	X	X	X
**6**		Gum Arabic	✓	X	X	X	X	X
**7**		Locust bean gum	✓	X	X	X	X	X
**8**		β-glucan	✓	X	X	X	X	X
**9**	Procyanidin B2	-	✓	✓	X	X	X	X
**10**		Xylobiose	✓	X	X	X	X	X
**11**		Cellobiose	✓	X	X	X	X	X
**12**		Monosaccharides	X	X	X	X	X	X
**13**		Uronic acids	✓	X	X	X	X	X
**14**	Epicatechin	-	X	X	X	X	X	X
**15**		Monosaccharides	X	✓	X	X	X	X
**16**		Uronic acids	X	X	X	X	X	X

***Structures below, numbers in parentheses refer to TFA adduct mass**

MONOMERS

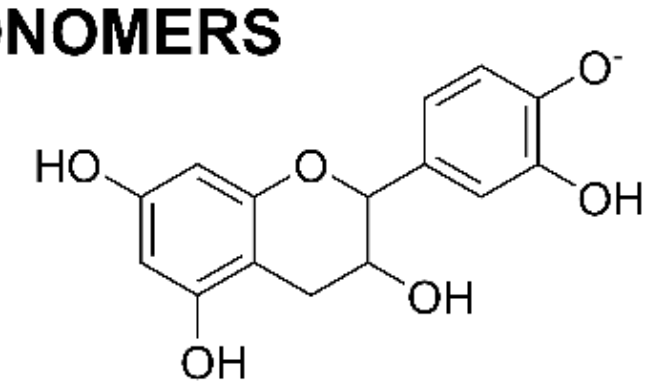

Chemical Formula: $C_{15}H_{13}O_6^-$
Molecular Weight: 289.3

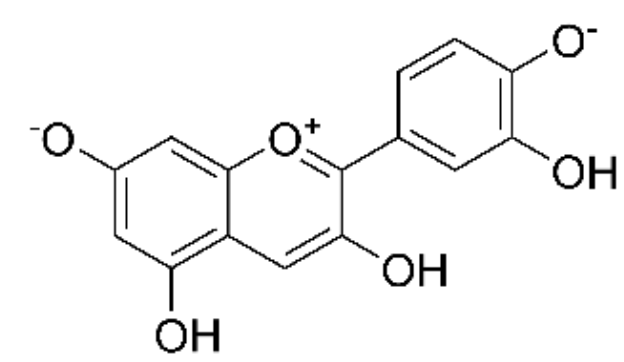

Chemical Formula: $C_{15}H_9O_6^-$
Molecular Weight: 285.2

ADDUCTS

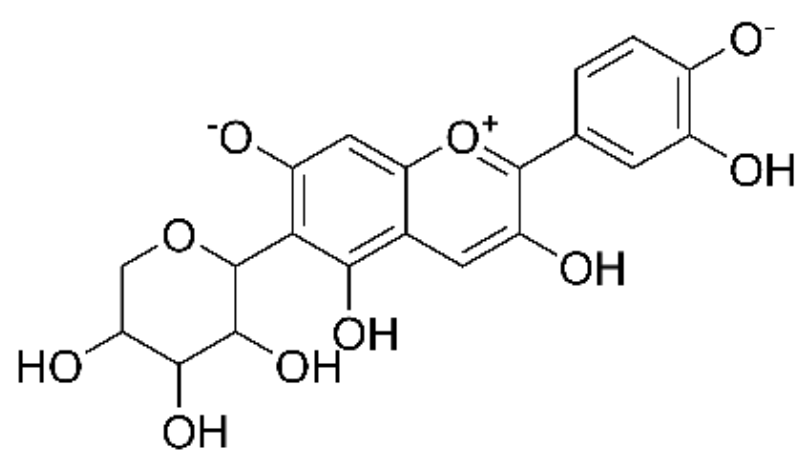

Chemical Formula: $C_{20}H_{17}O_{10}^-$
Molecular Weight: 417.3

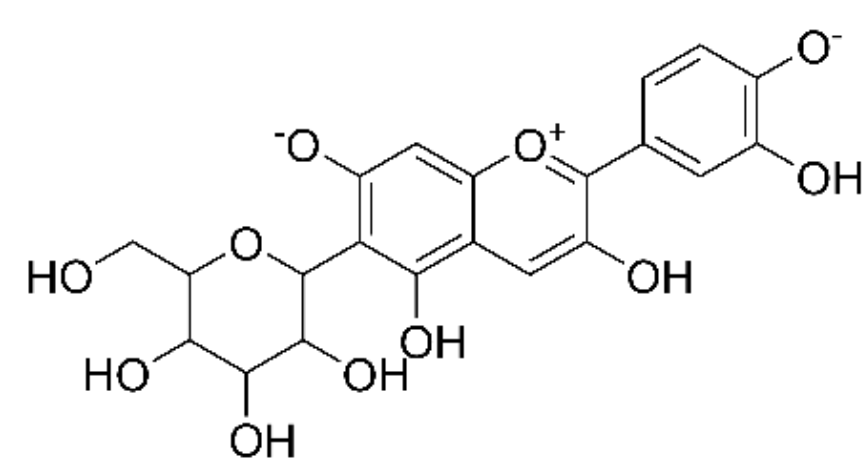

Chemical Formula: $C_{21}H_{19}O_{11}^-$
Molecular Weight: 447.4

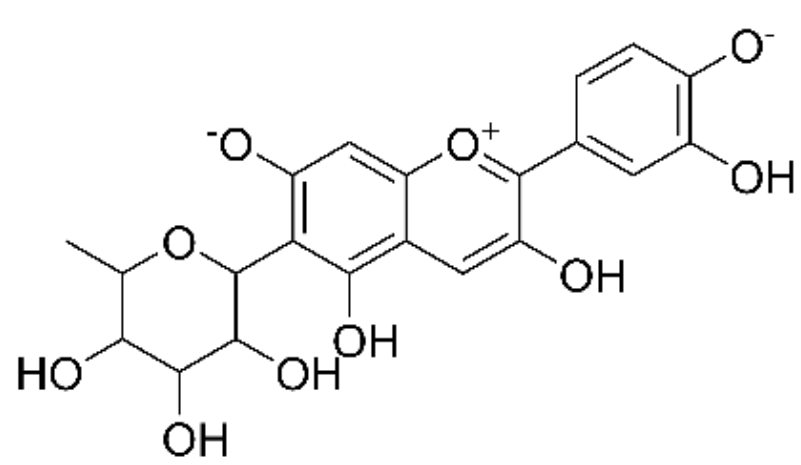

Chemical Formula: $C_{21}H_{19}O_{10}^-$
Molecular Weight: 431.4

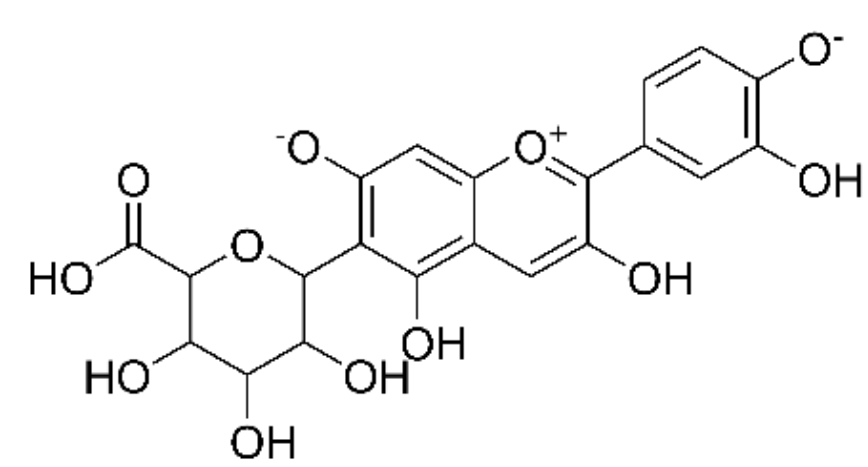

Chemical Formula: $C_{21}H_{17}O_{12}^-$
Molecular Weight: 461.4

Table S4. The yield of anthocyanidins after acid butanol hydrolysis of AIR and subsequent purification.

Anthocyanidin	mmol/g AIR
Delphinidin (in crude)	1×10^{-4}
Cyanidin	7×10^{-5}
Pelargonidin	8×10^{-7}
Peonidin	2×10^{-6}
**Total yield, mmol/g**	**2×10^{-4}**

Table S5. Chemical shift assignments of depolymerized WRC bark AIR based on HSQC and HMBC spectra.

	δ_H, ppm	δ_C, ppm
Cyanidin		
2	-	160.06
3	-	145.66
4	8.61	133.08
5	-	155.24
6	6.86	94.02
7	-	167.39
8	6.70	102.44
9	-	157.97
10	-	112.52
1’	-	120.75
2’	8.05	117.07
3’	-	146.39
4’	-	153.72
5’	7.02	116.60
6’	8.10	125.66
C-pentose		
1”	4.70	71.75
2”		
3”		
4”		
5”		68.75

III. Supplementary Figures

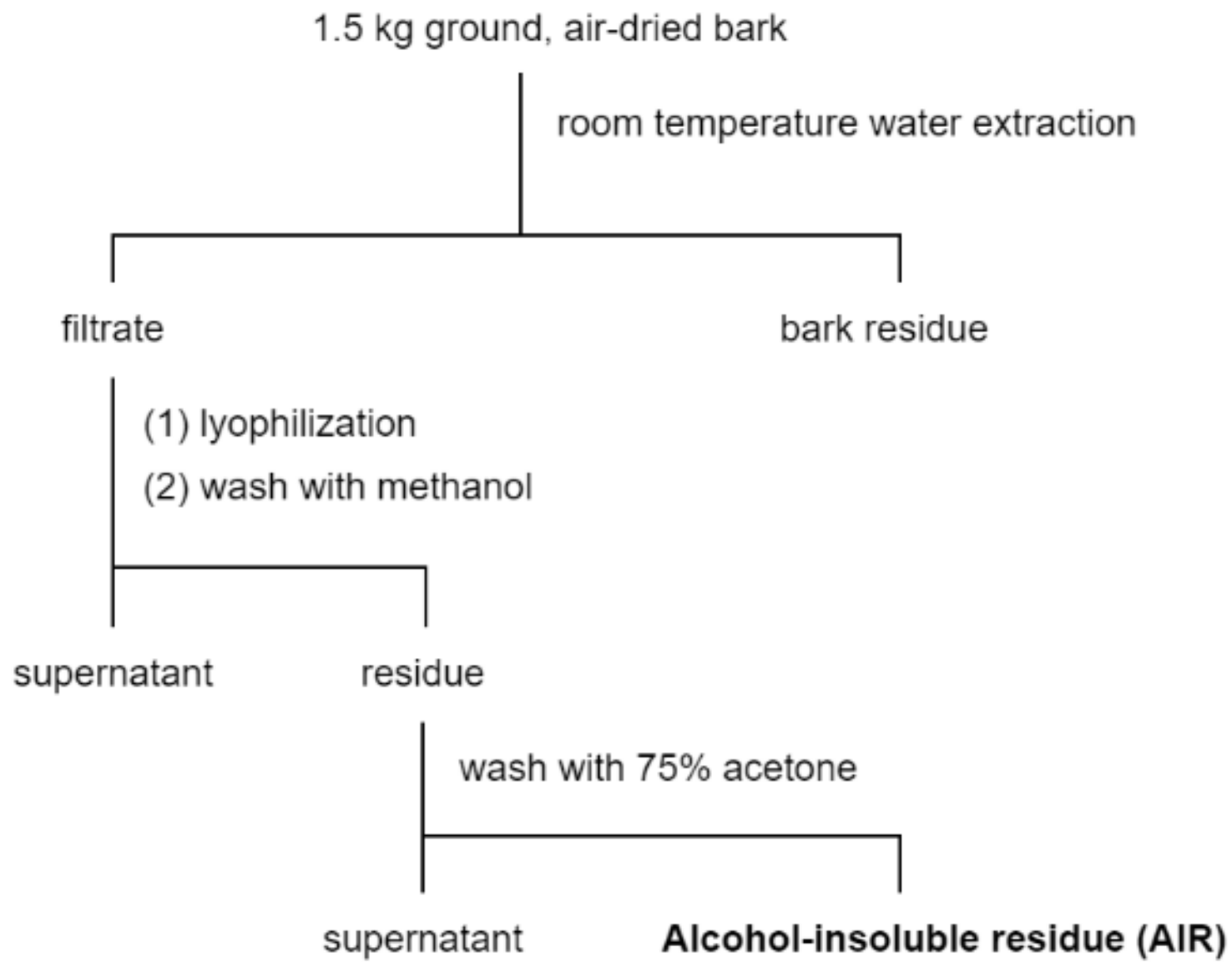

Figure S1. Scheme for the preparation of WRC bark AIR.

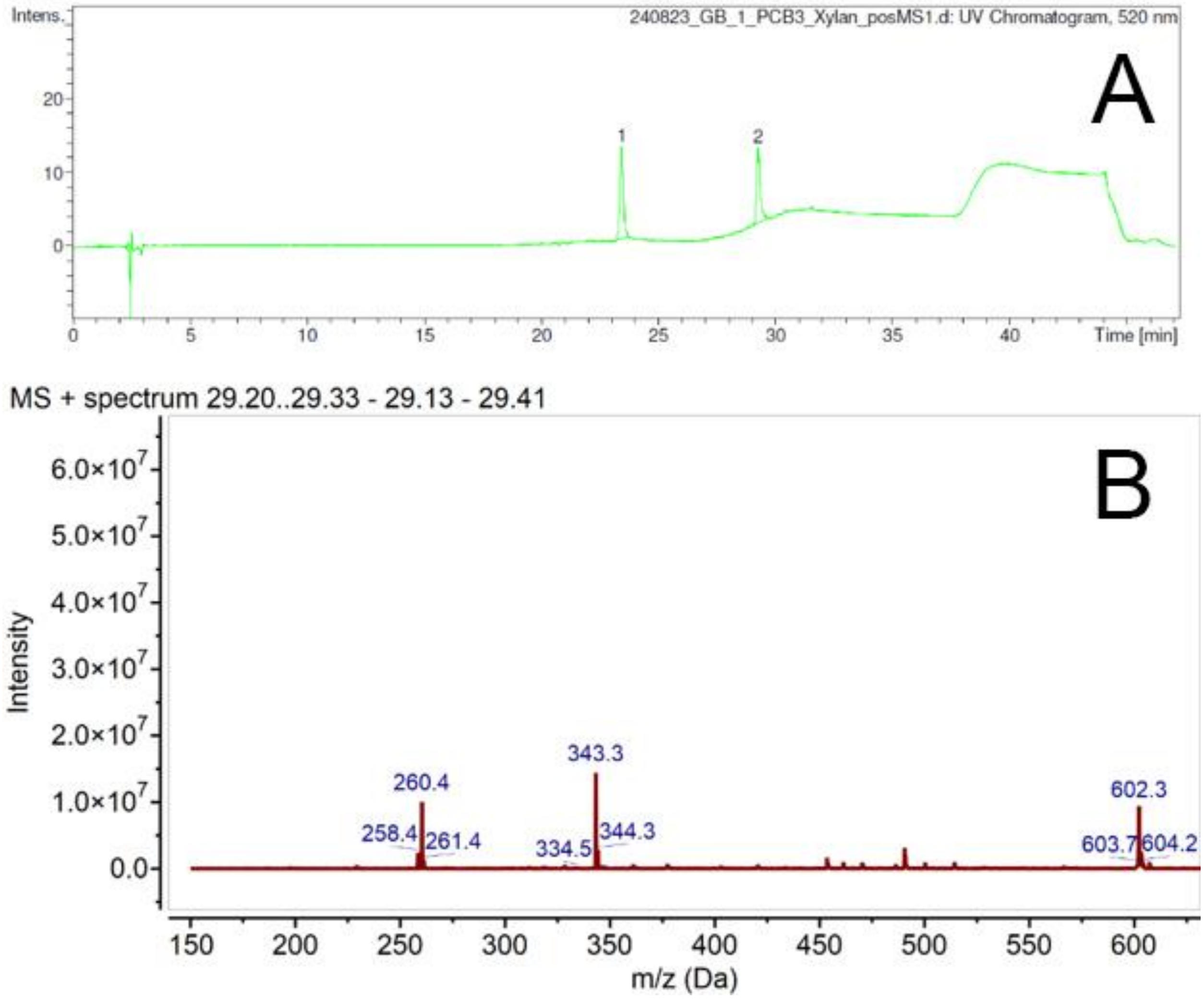

Figure S2. Representative chromatogram for the analysis of anthocyanidins in test controls (A). Peak 1 is cyanidin, Peak 2 is butylated cyanidin. Mass spectrum for peak 2 is shown, with mass of 343.3 *m/z* (B).

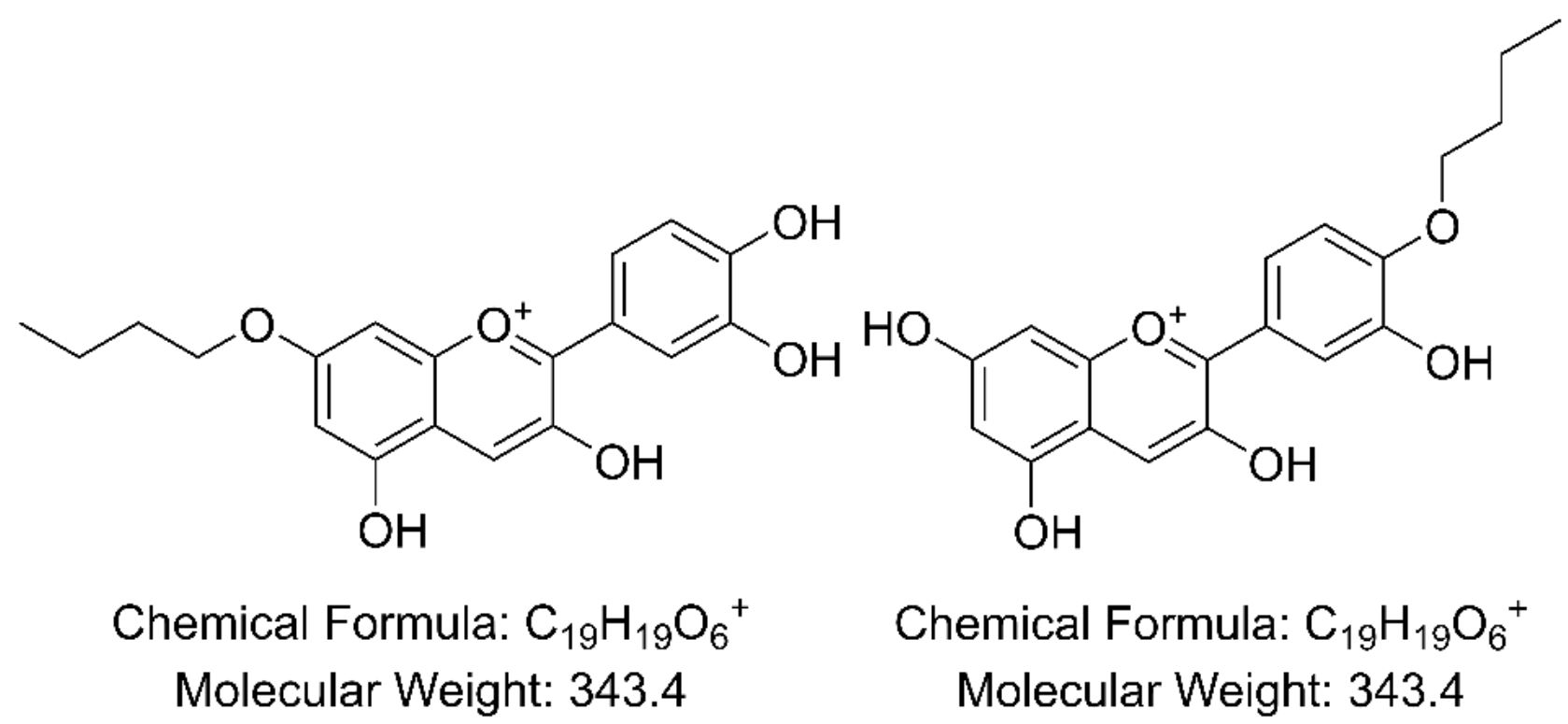

Figure S3. Hypothetical structures for cyanidin-butanol adducts.

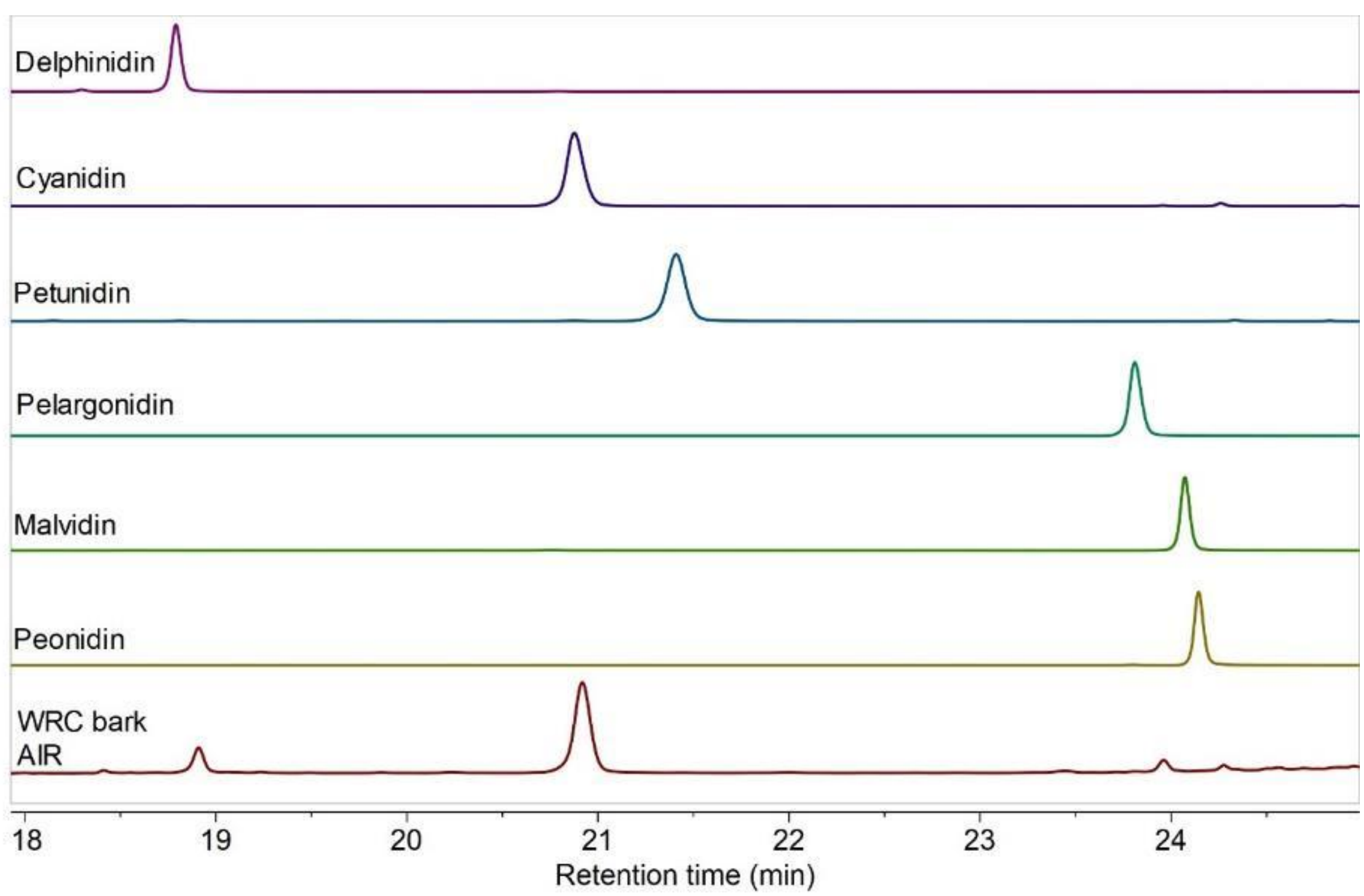

Figure S4. Detected anthocyanidins after acid butanol hydrolysis of WRC bark AIR.

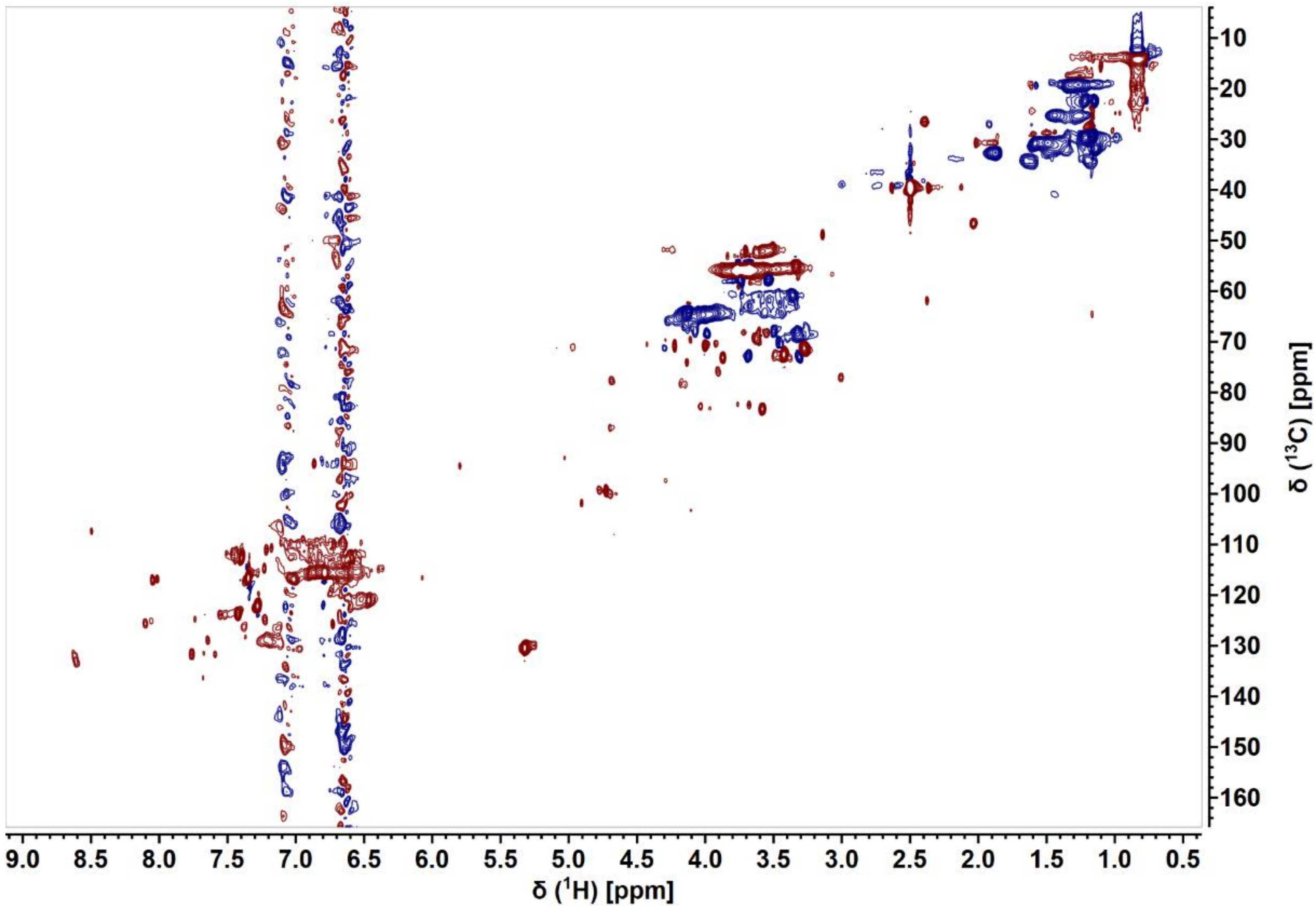

Figure S5. HSQC of depolymerized WRC bark AIR.

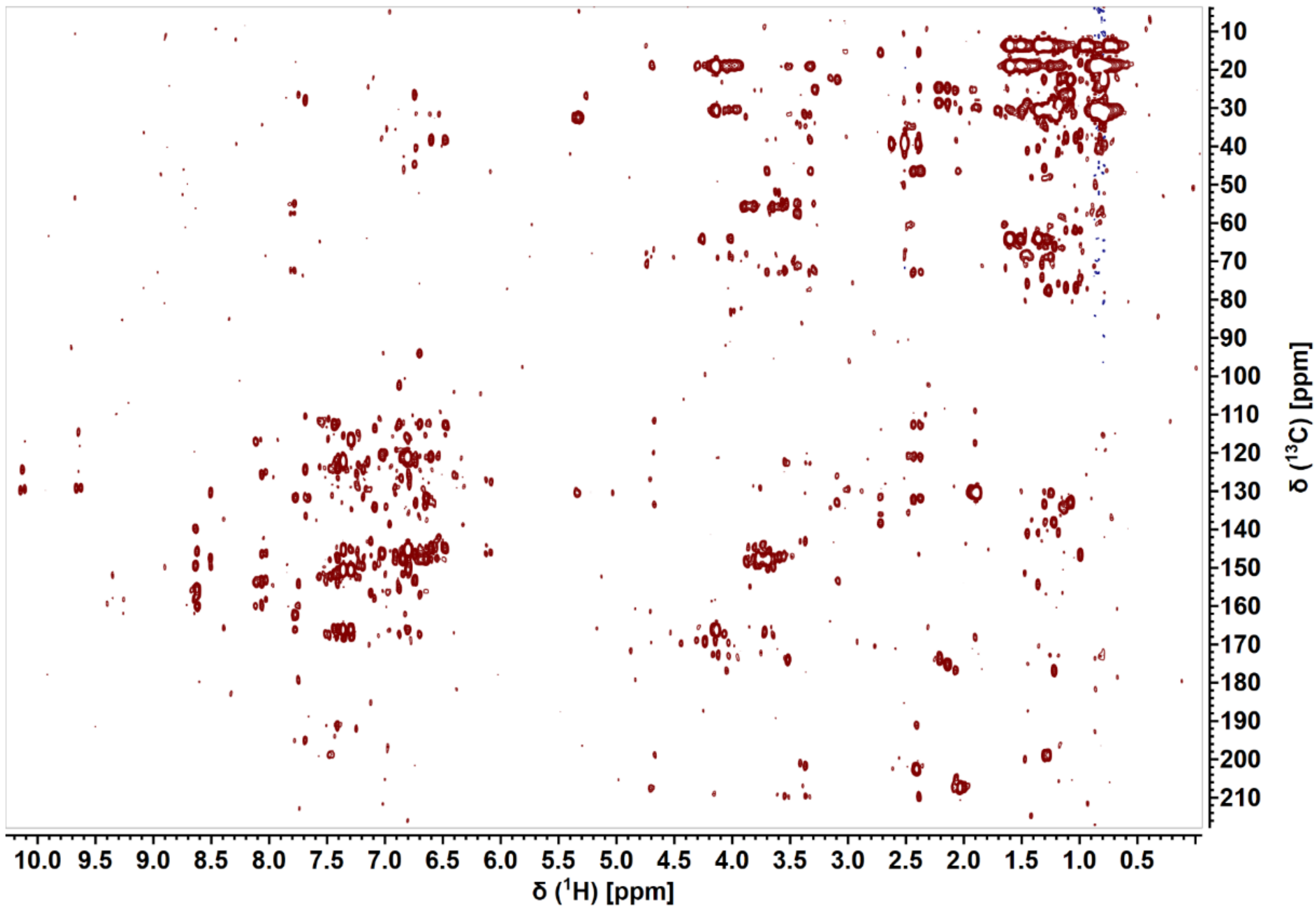

Figure S6. HMBC of depolymerized WRC bark AIR.

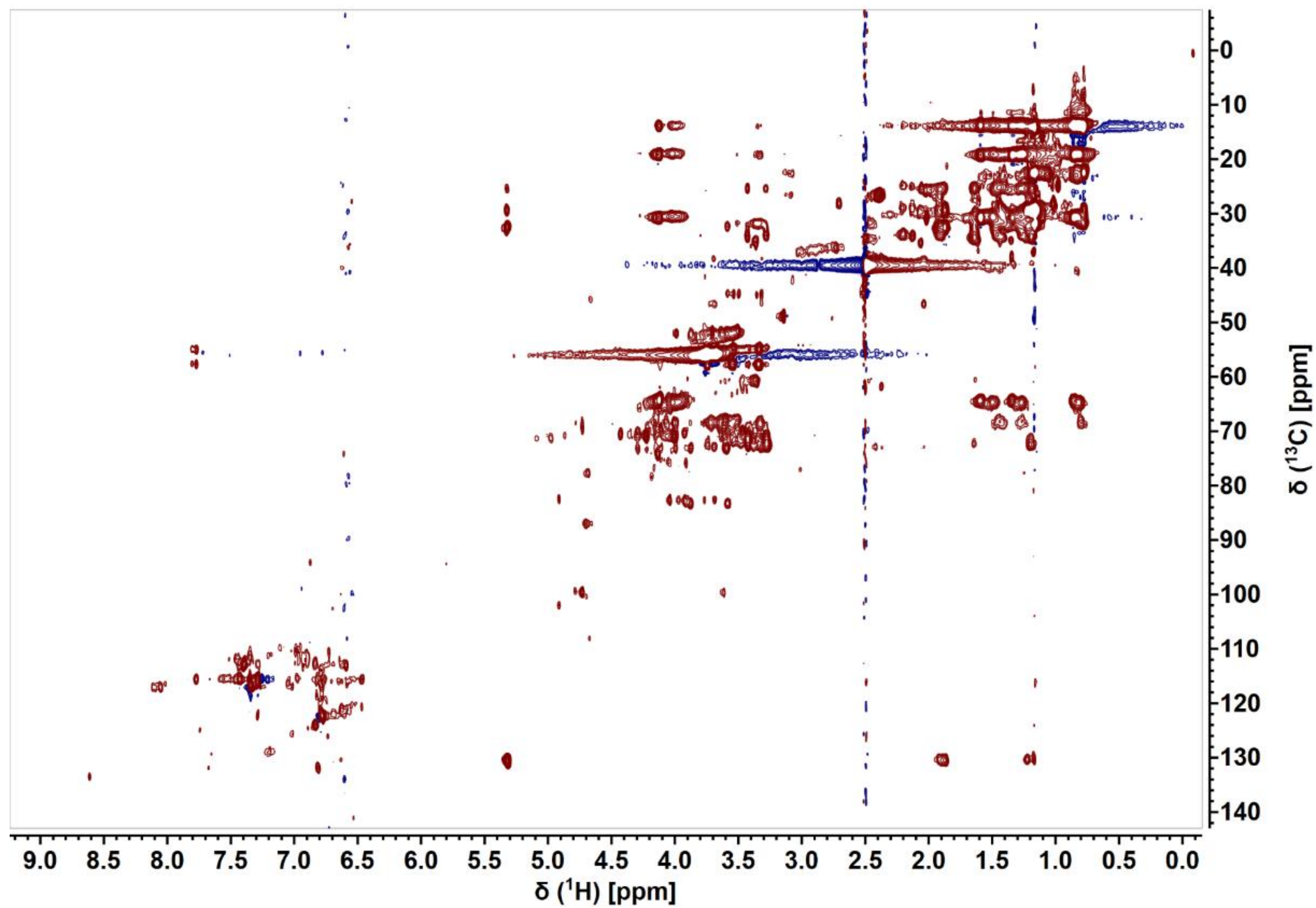

Figure S7. HSQC-TOCSY of depolymerized WRC bark AIR.

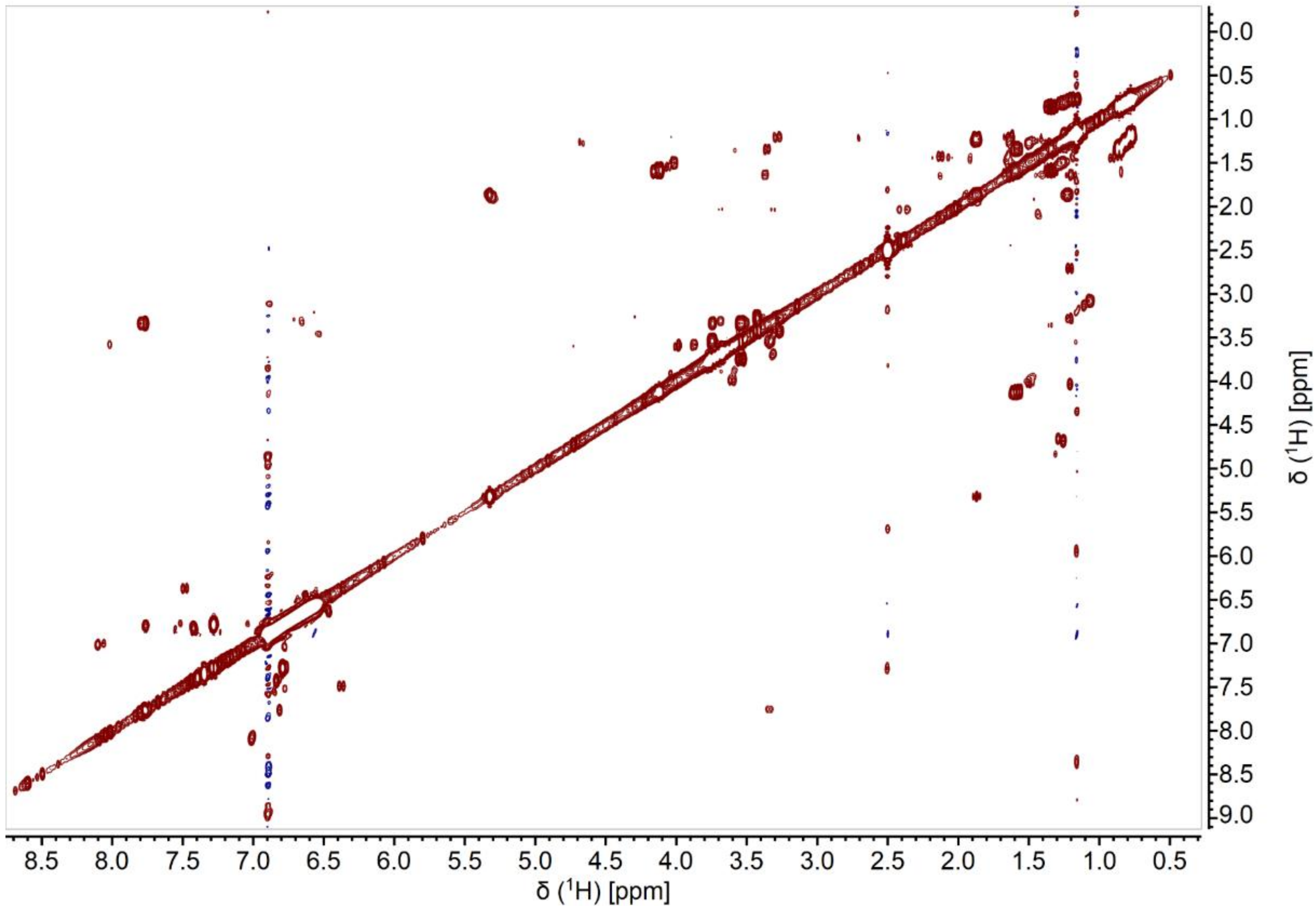

Figure S8. COSY of depolymerized WRC bark AIR.

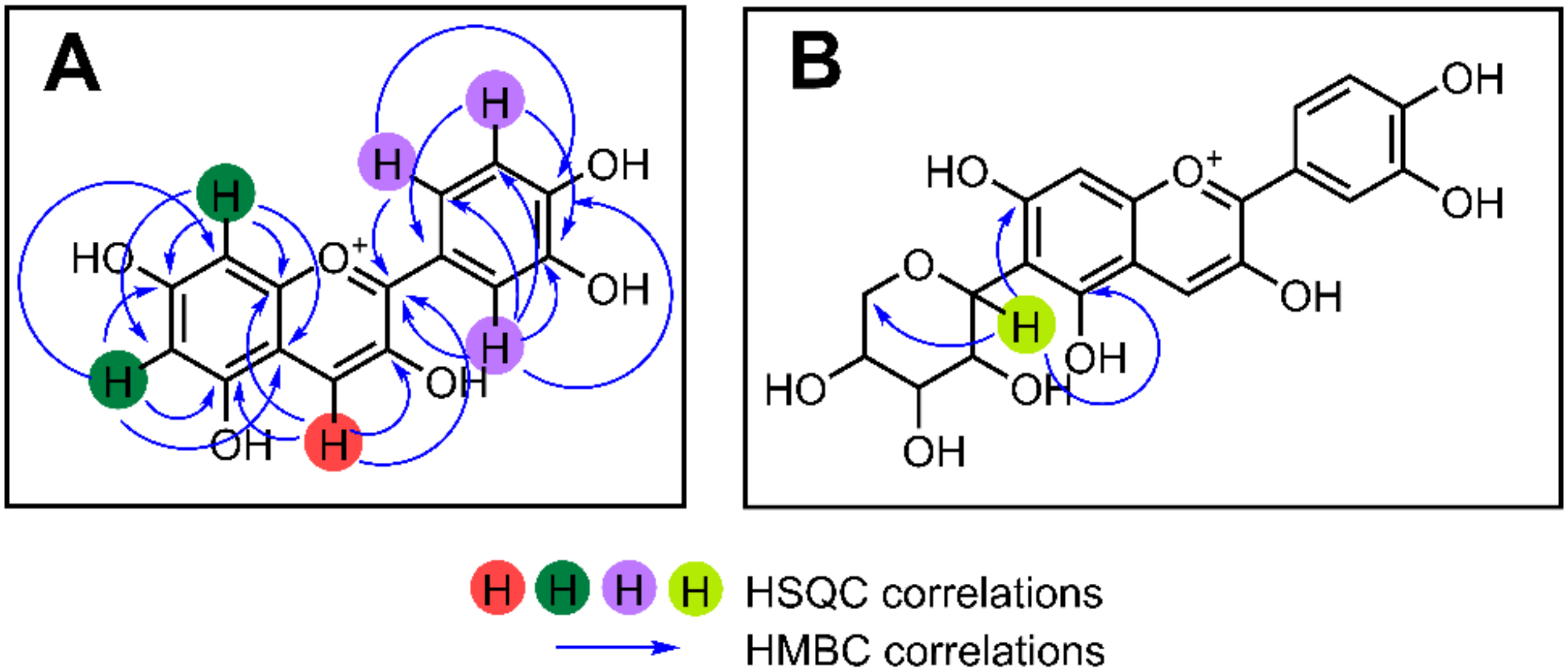

Figure S9. Summary of key NMR correlations for cyanidin nucleus (A), and the proposed *C*-glycosylanthocyanins in WRC bark AIR.

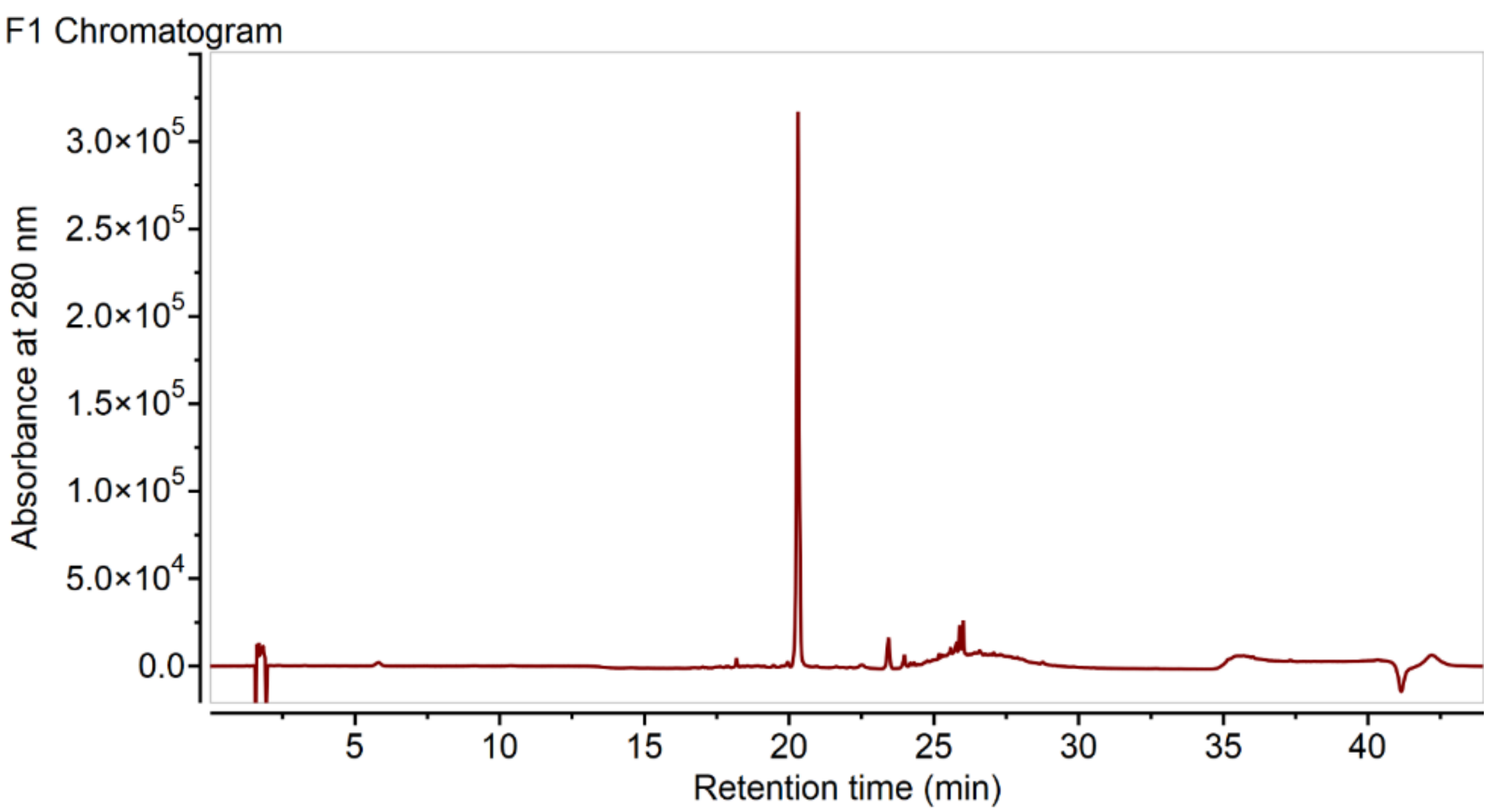

Figure S10. UV trace at 280 nm of Fraction F1.

Scheme S1. Fragmentations for 416 *m/z*

<u>Positive ion mode</u>

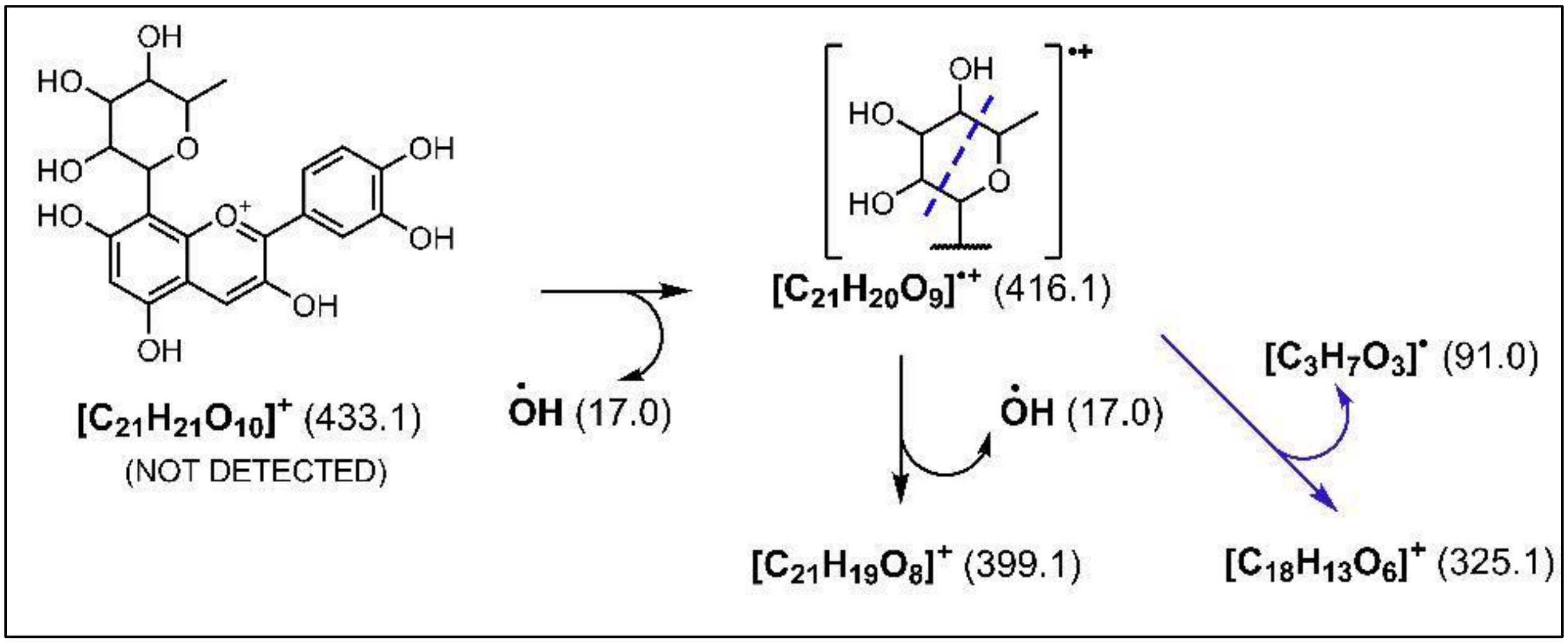

<u>Negative ion mode</u>

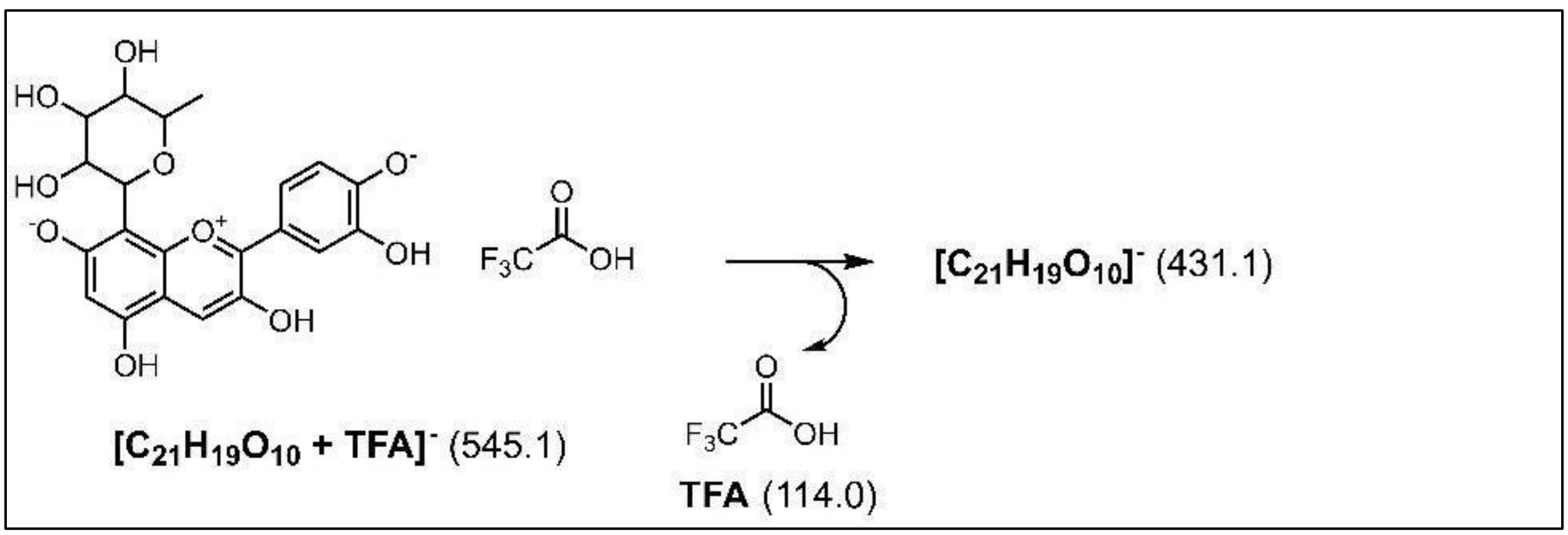

Scheme S2. Fragmentations for 419 *m/z*

Positive ion mode

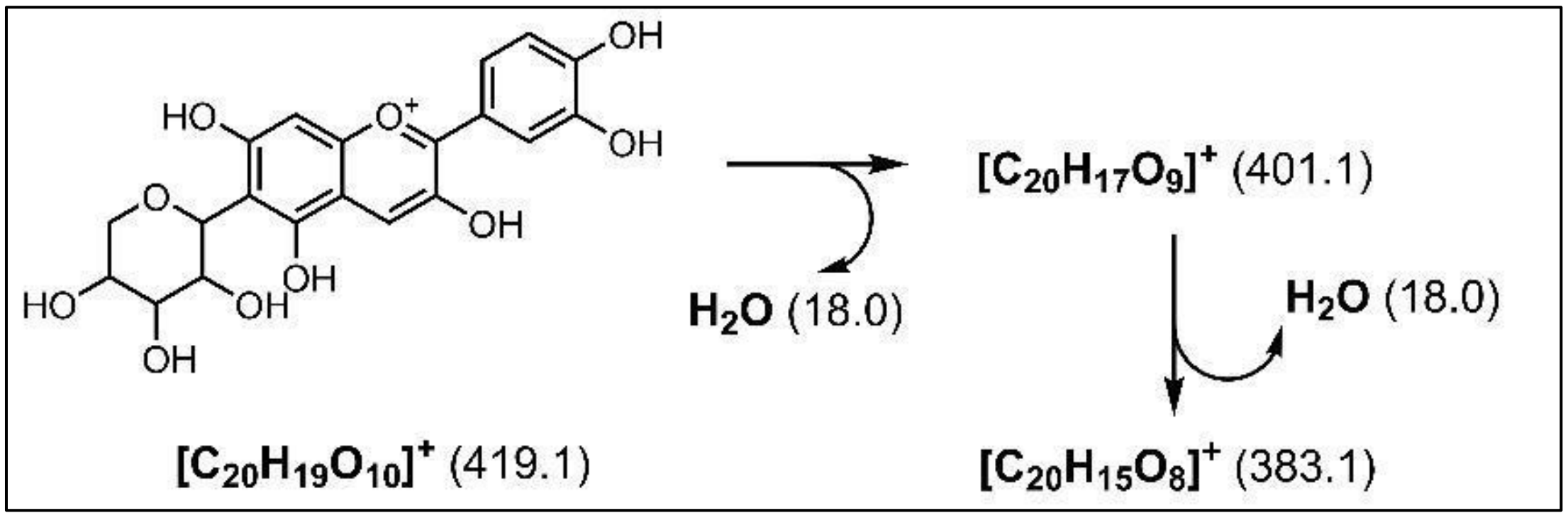

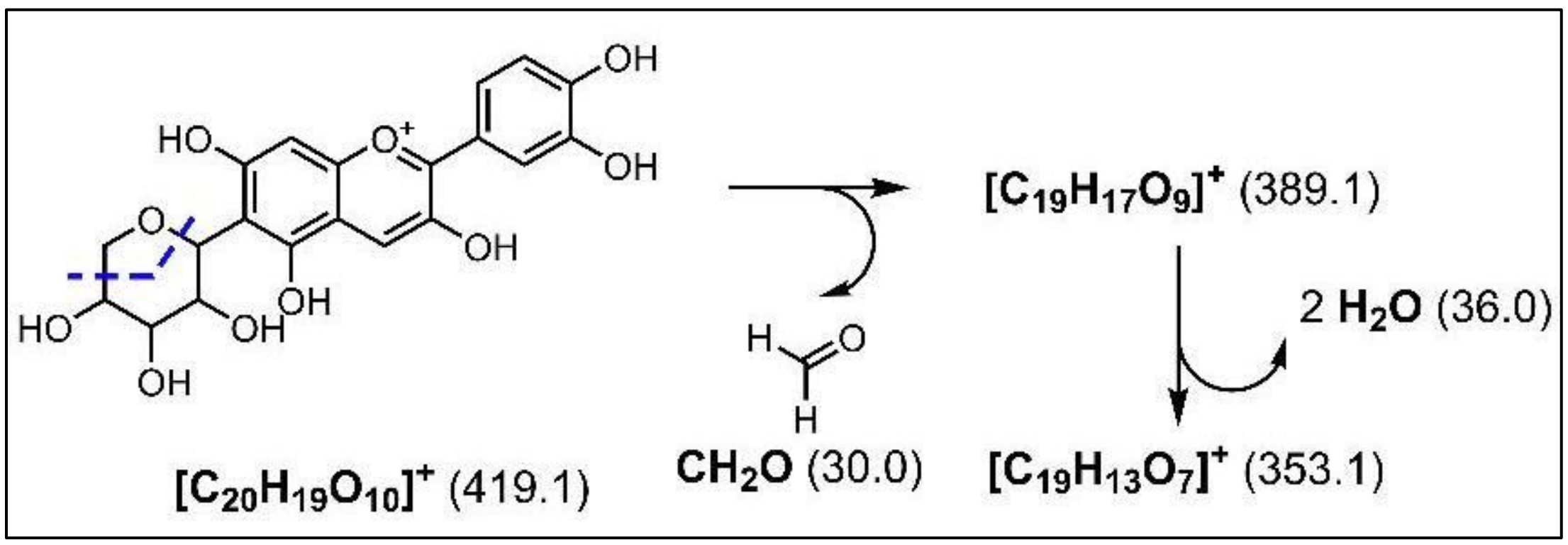

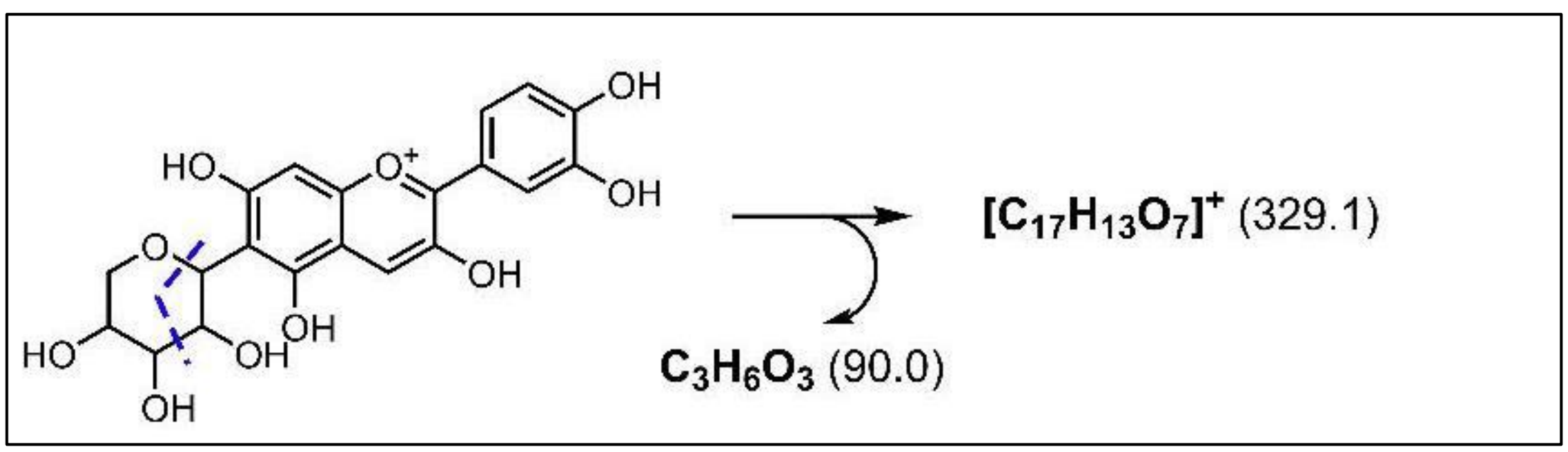

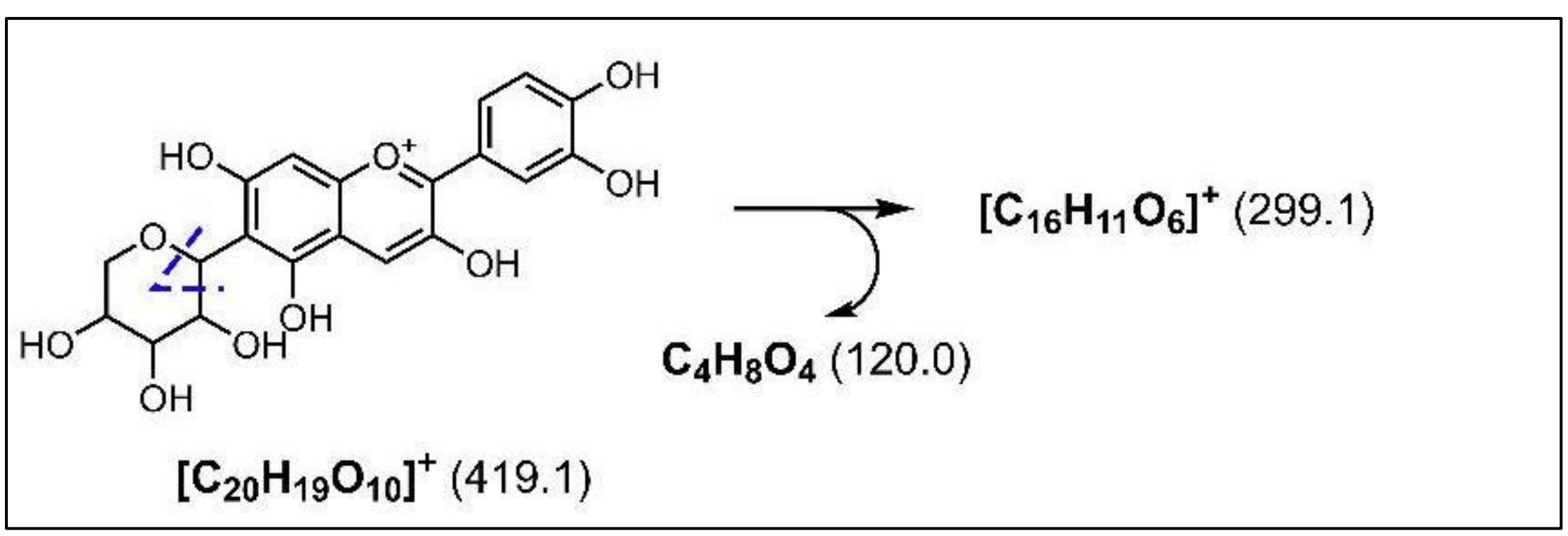

Negative ion mode

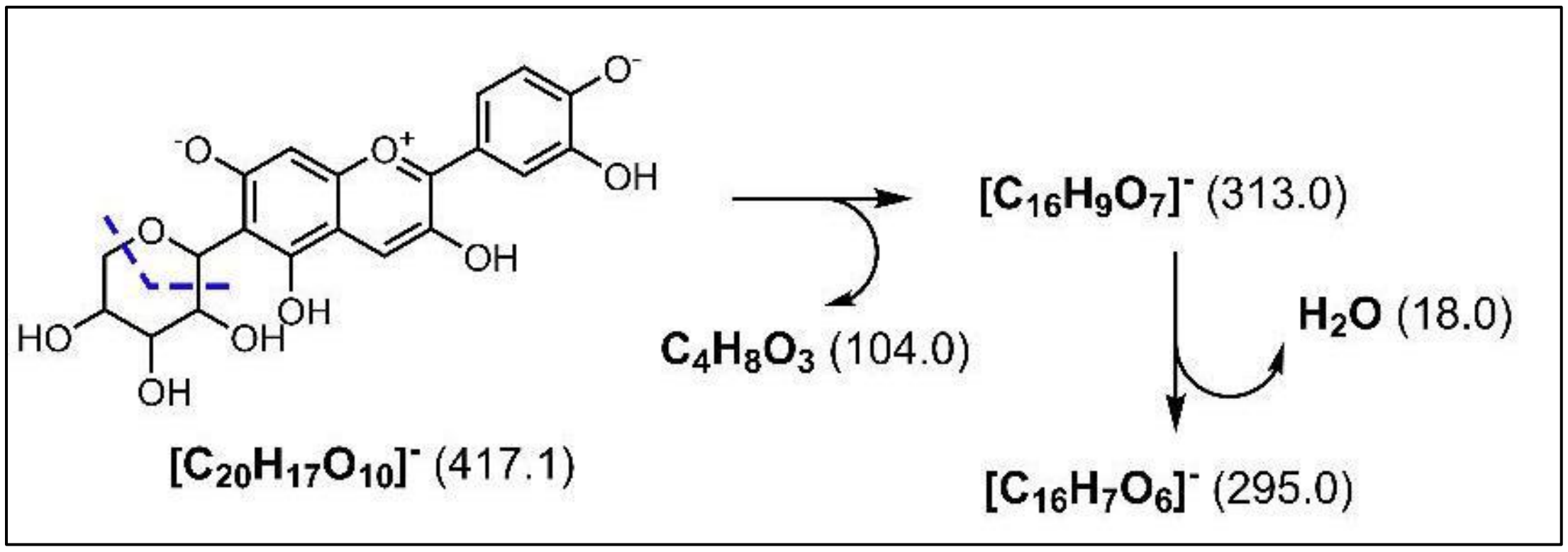

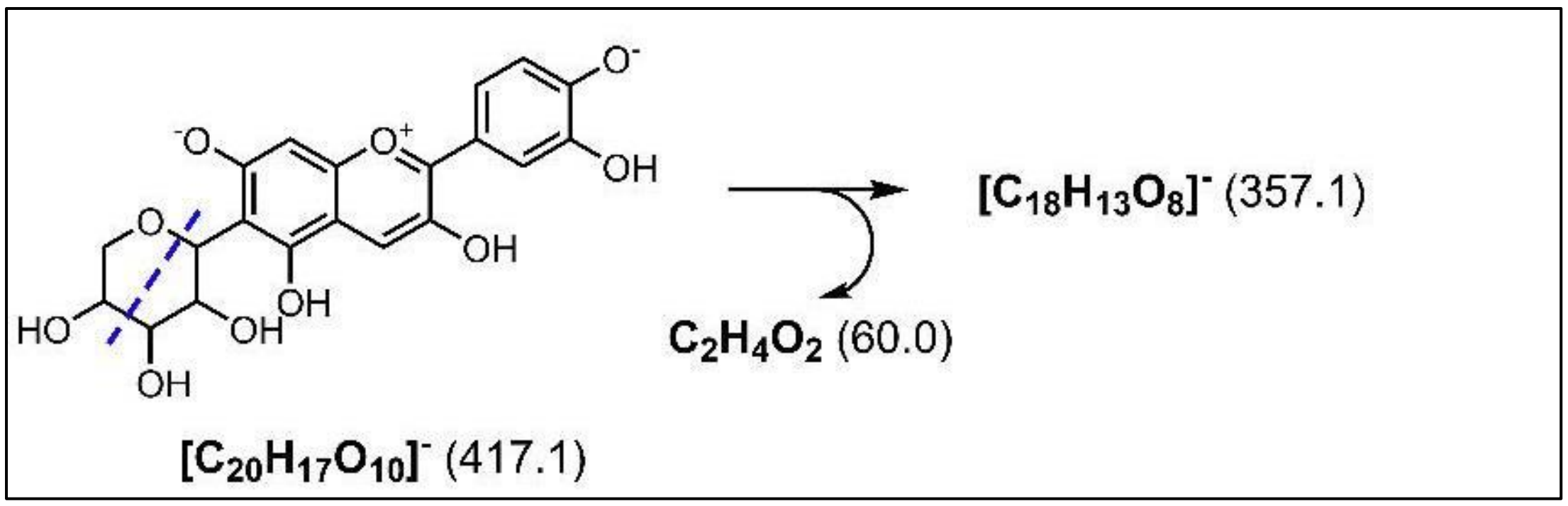

Scheme S3. Fragmentations for 599 *m/z*

__Positive ion mode__

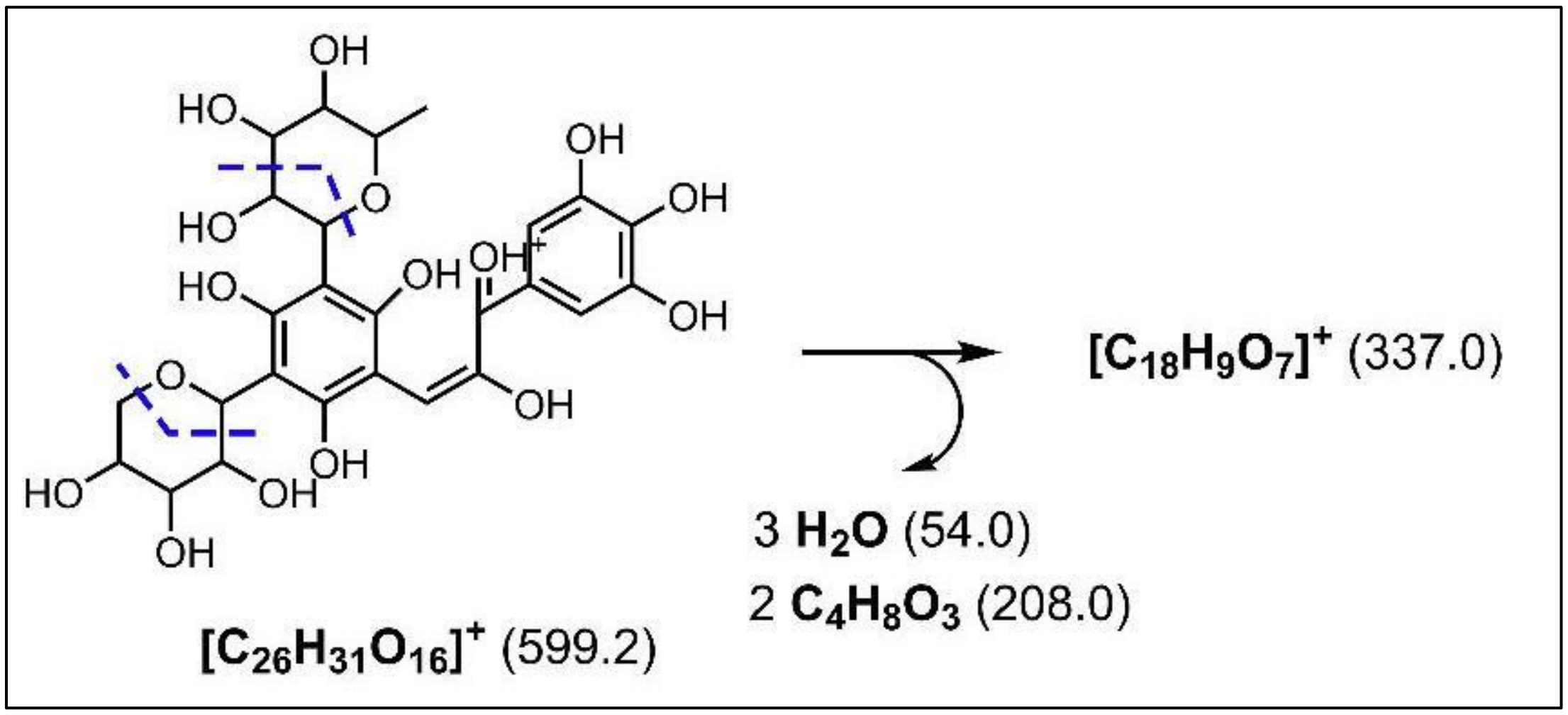

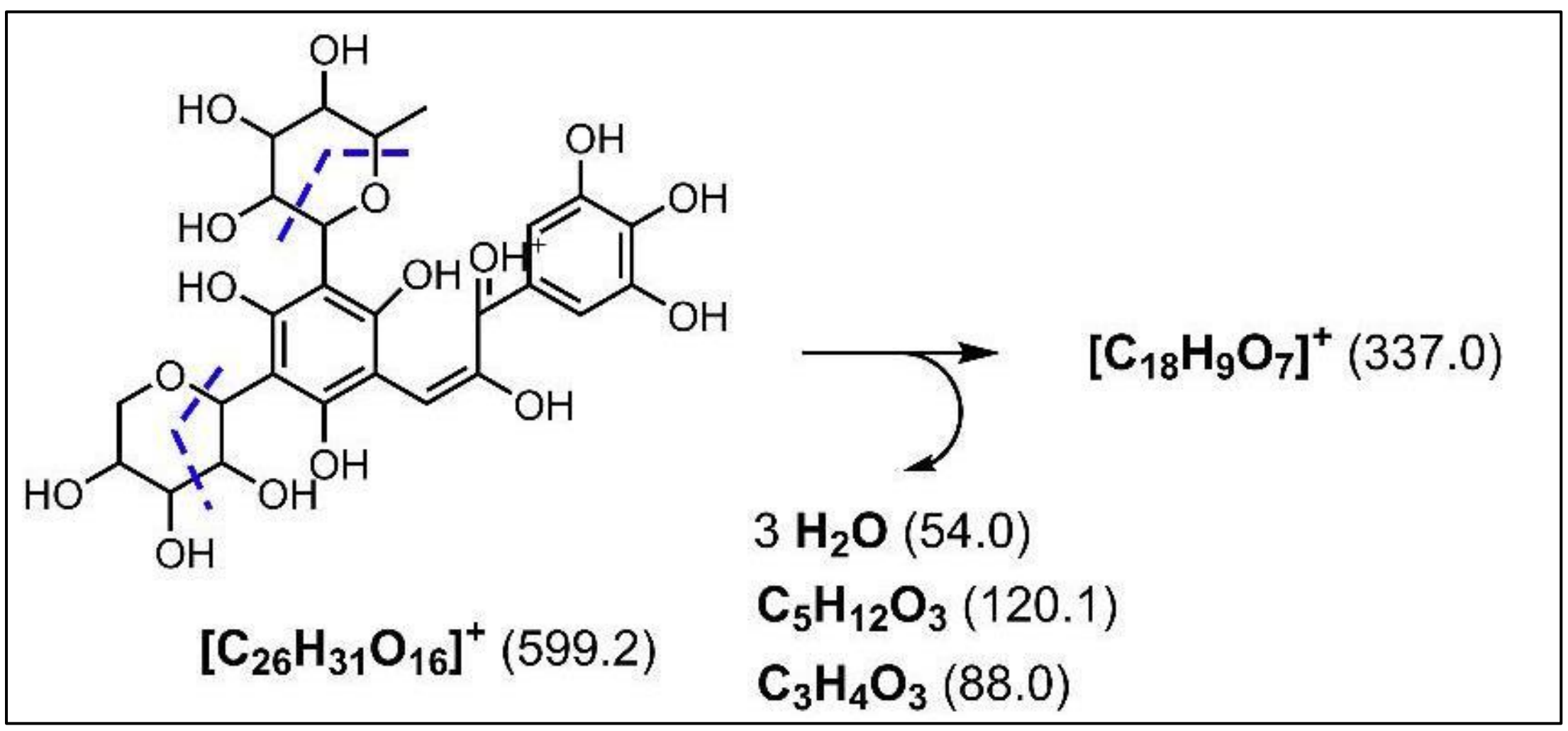

Negative ion mode

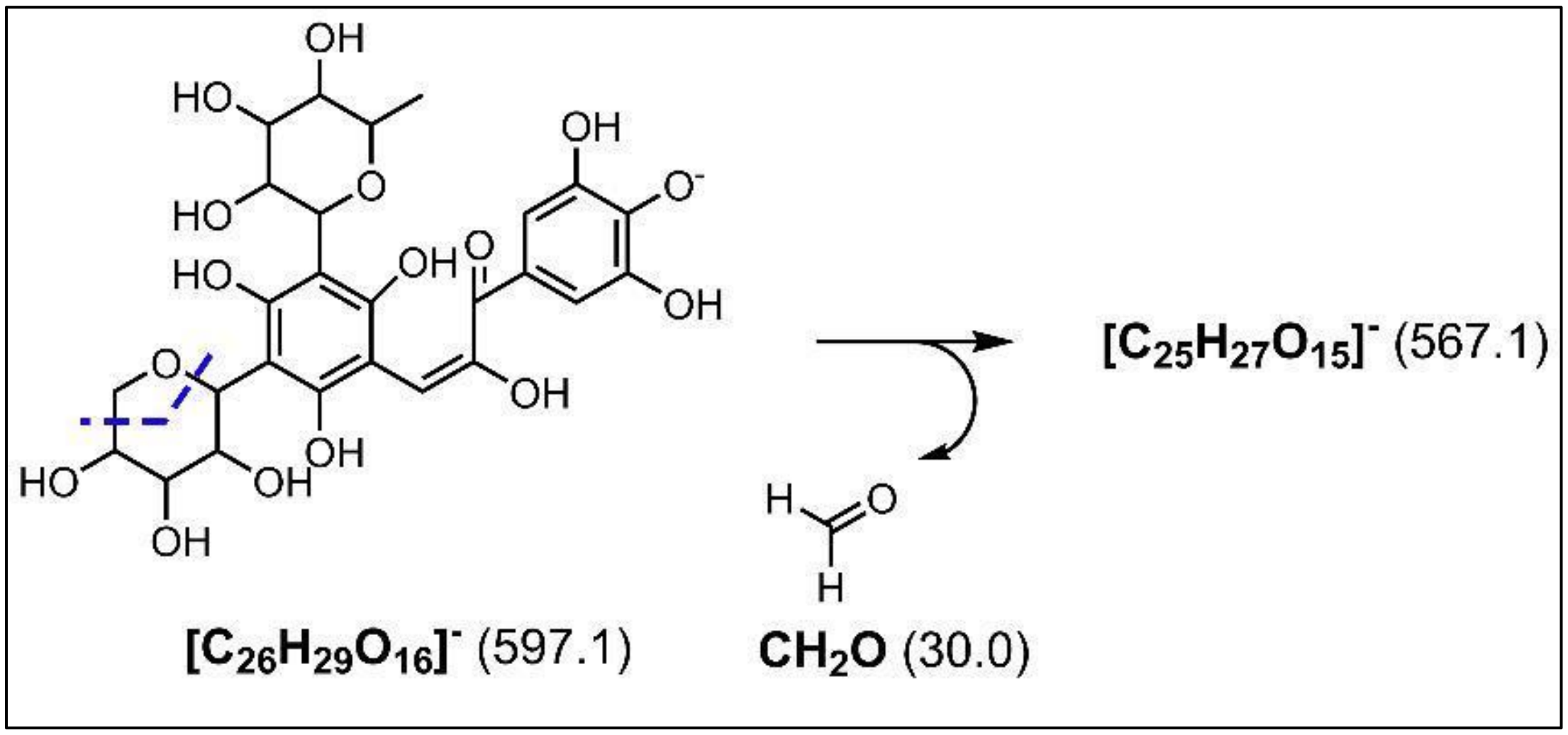

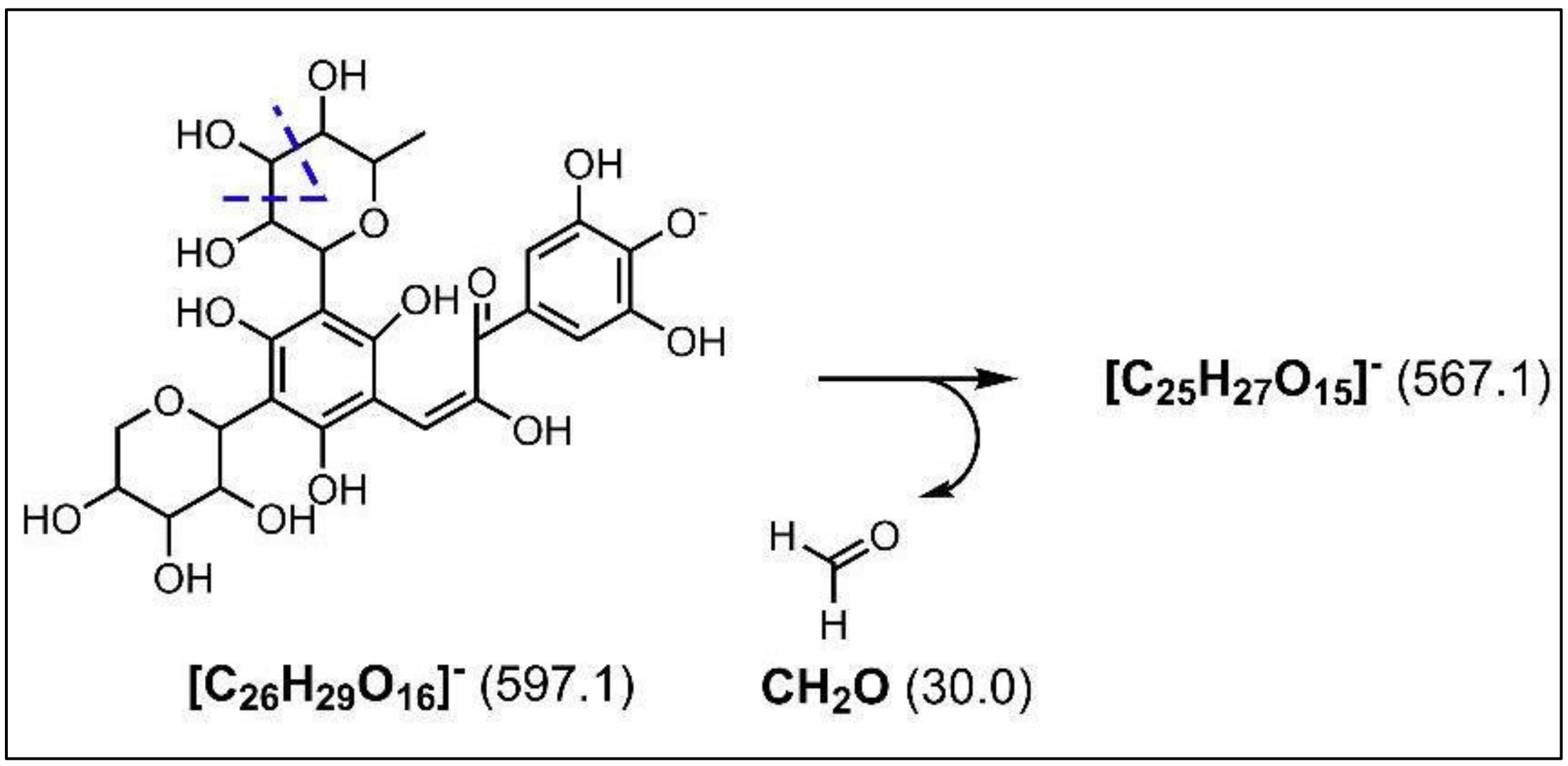

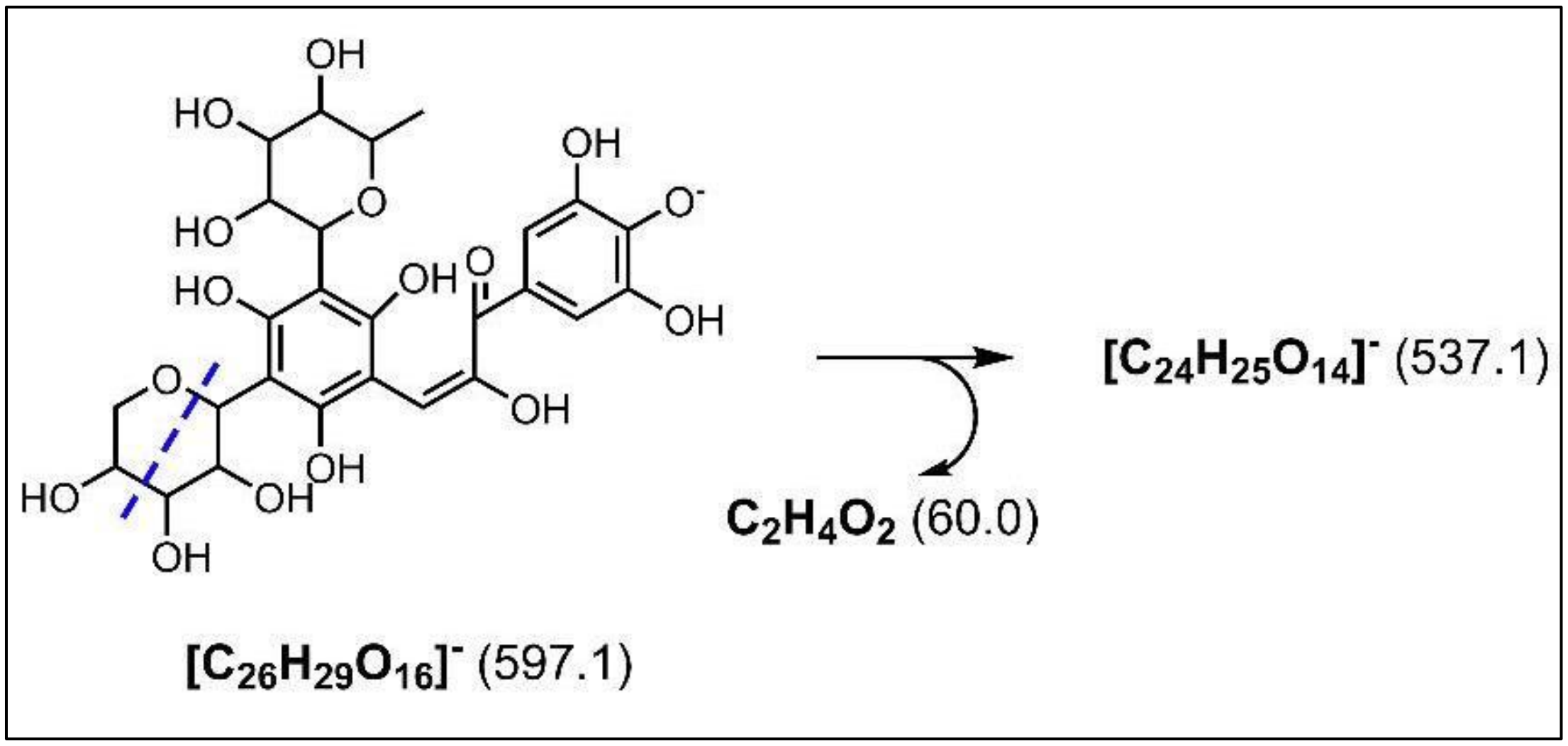
[C26H29O16]- (597.1)
[C24H25O14]- (537.1)
C2H4O2 (60.0)

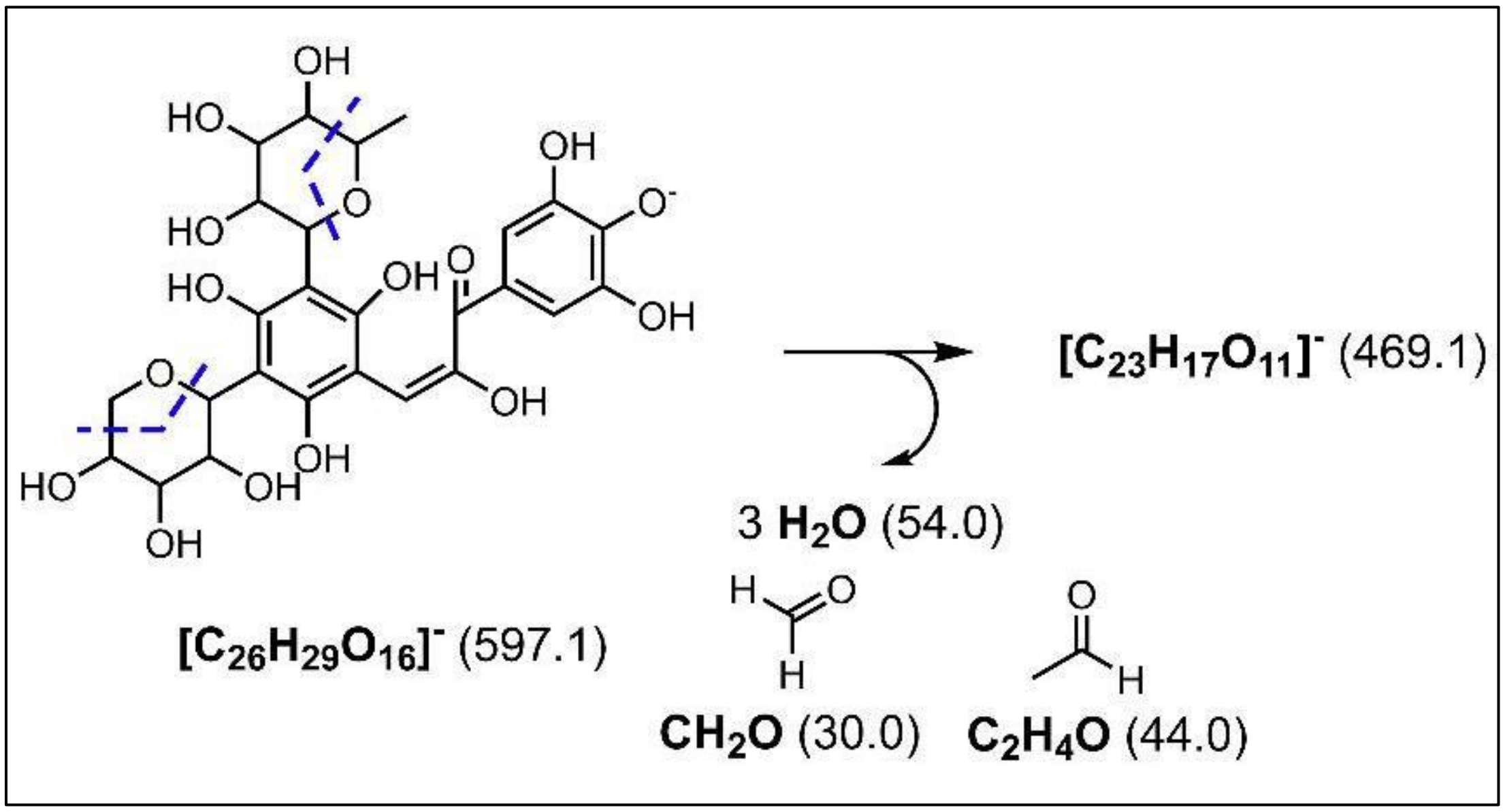
[C26H29O16]- (597.1)
[C23H17O11]- (469.1)
3 H2O (54.0)
CH2O (30.0)
C2H4O (44.0)

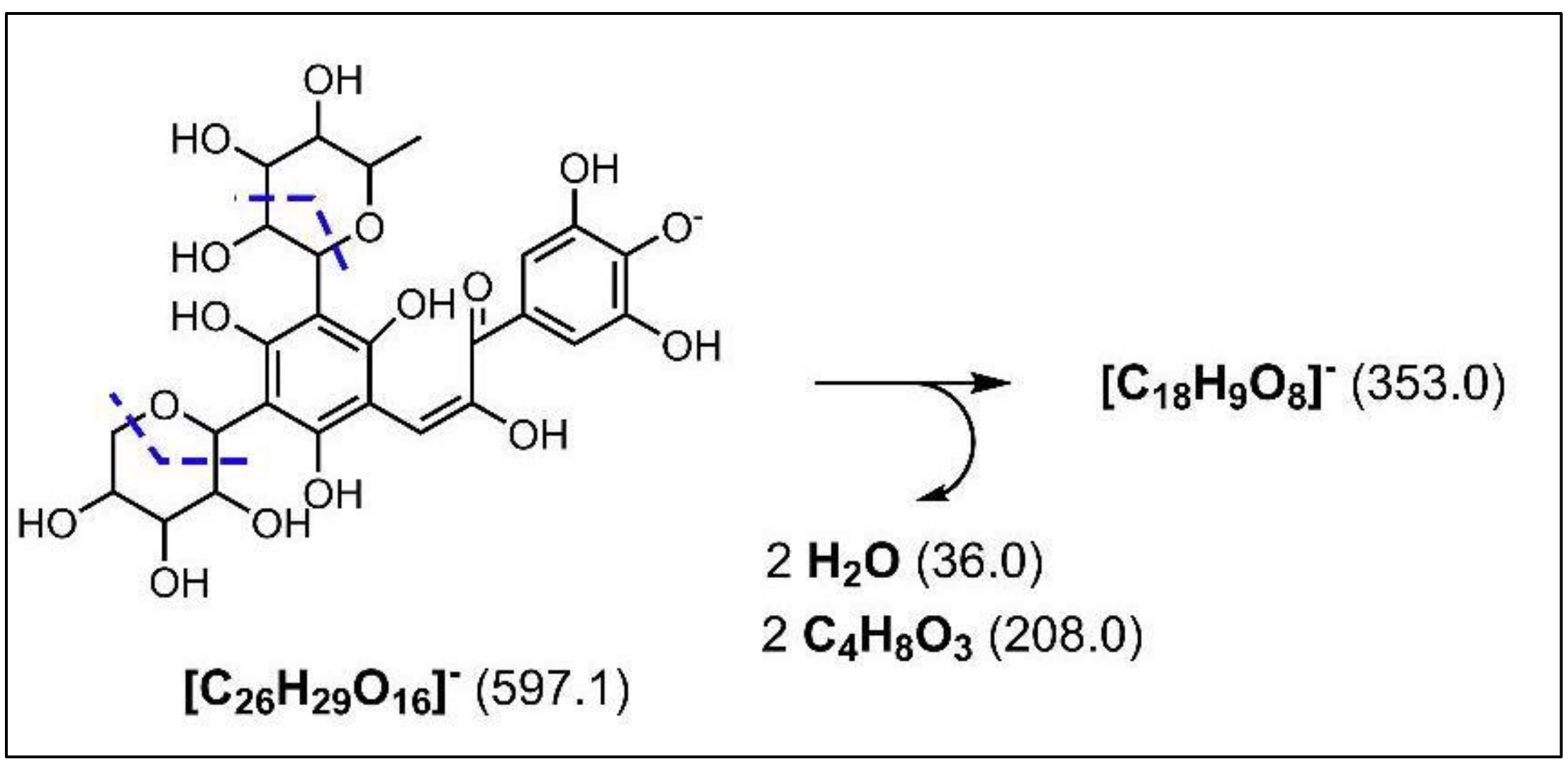
[C26H29O16]- (597.1)
[C18H9O8]- (353.0)
2 H2O (36.0)
2 C4H8O3 (208.0)
